# Unraveling potential EGFR kinase inhibitors: Computational screening, molecular dynamics insights, and MMPBSA analysis for targeted cancer therapy development

**DOI:** 10.1371/journal.pone.0321500

**Published:** 2025-05-09

**Authors:** Muhammad Naseem Khan, Umar Farooq, Aneela Khushal, Tanveer A. Wani, Seema Zargar, Sara Khan

**Affiliations:** 1 Department of Chemistry, COMSATS University Islamabad, Abbottabad Campus, Abbottabad, Pakistan; 2 Beijing National Laboratory for Molecular Sciences, State Key Laboratory of Molecular Reaction dynamics, Institute of Chemistry, Chinese Academy of Sciences, Beijing, China; 3 Department of Pharmaceutical Chemistry, College of Pharmacy, King Saud University, Riyadh, Saudi Arabia; 4 Department of Biochemistry, College of Science, King Saud University, Riyadh, Saudi Arabia; 5 Department of Chemistry, Pennsylvania State University, University Park, Pennsylvania, United States of America; University of Mashreq, IRAQ

## Abstract

EGFR is critical for tumor angiogenesis and cancer progression, but existing treatments like erlotinib face limitations such as acquired resistance and side effects. To address these issues, this study employs structure-based drug design techniques including virtual screening, molecular docking, and molecular dynamics simulations to identify new small molecule inhibitors targeting the EGFR kinase domain. From an initial selection of 633,000 compounds from diverse databases, top candidates were identified based on their binding affinity and stability. The virtual screening and docking analyses revealed compounds with higher binding scores than erlotinib. Molecular dynamics simulations and Anisotropic Network Model (ANM) analysis uniquely report that EGFR undergoes significant conformational shifts: inward flap movements in the bound state stabilize a closed conformation, while outward movements in the free state result in a more open conformation. Among the identified inhibitors, compounds such as JFD00243, NPA015124, and others exhibited strong binding affinities and stable interactions with both active and inactive forms of EGFR. Notably, JFD00243 was effective in targeting EGFR in both active and inactive conformations. These findings suggest that the identified inhibitors could potentially overcome current treatment limitations and improve targeted cancer therapies by effectively inhibiting EGFR-mediated tumor angiogenesis.

## Introduction

Targeted therapies, in addition to conventional cancer treatments, have gained significant interest in the recent past [1–3]. These therapies mainly focus on important biomolecules that are crucial either for the normal physiological cellular functioning, replication, or tumor development. They have the tendency to bring cytostatic and/or cytotoxic effects on affected cells while reducing the non-specific toxicities linked to radiation or chemotherapy [4].

Epidermal growth factor (EGFR) is a type of receptor tyrosine kinase enzyme which functions as a transmembrane glycoprotein [5]. The signaling pathway involving the EGFR is among the most crucial pathways within mammalian cells [6]. Certain ligands, such as epidermal growth factor (EGF) and transforming growth factor *alpha* (TGFα), engage with and stimulate EGFR, leading to the autophosphorylation of EGFR tyrosine kinase domain and catalyzing the phosphorylation of tyrosine residues of the target proteins by transferring the phosphate group from an ATP molecule [7,8]. The phosphorylated proteins lead to signal transduction. The active EGFR protein is responsible for various signal transductions in normal processes of cell proliferation and DNA synthesis [9,10].

Cancer is caused when DNA is mutated leading to abnormal cell proliferation [11,12]. Mutations in EGFR are major reasons for initiation of abnormal cell division [13,14]. These mutations result in the overexpression of the proteins, thereby, activating various downstream pathways in undefined manner followed by inception of different malignancies, particularly non-small cell lung cancer (NSCLC) [15], which constitutes around 75% of all reported lung cancers. Therefore, EGFR is considered as a key target in combating such uncontrolled proliferation in cancer therapy. For cancer to be effectively treated, inhibitors that target both the active and inactive forms of EGFR protein conformations must be developed. The active form of EGFR facilitates the ATP binding and kinase activity whereas the inactive form adopts conformation influencing signaling. Inhibitors targeting the active EGFR block the kinase activity and mutations often confer resistance, therefore requiring alternative approaches. Hence, targeting the inactive conformation of EGFR presents an opportunity to overcome this problem by stabilizing the EGFR in its inactive conformation. Finding inhibitors against both forms of EGFR increases the therapeutic scope ensuring comprehensive inhibition, minimizing resistance and tumor progression. Currently, different drugs including Erlotinib, Afatinib, Gefitinib, Icotinib, and Brigatinib (S1 Fig) are available as EGFR protein inhibitors in the market.

The significance of computational predictions in the field of biology and biomedicine cannot be overstated, as they offer efficient means of obtaining valuable data that would otherwise require extensive time and resources to acquire solely through experimental methods [16–21]. When combined with insights obtained from structural bioinformatics analysis, these computational predictions can furnish timely and valuable information for both fundamental scientific research and the development of pharmaceuticals [22,23]. Indeed, many groundbreaking advancements in cancer drug development have hinged on the utilization of computational bioinformatics and modeling techniques [24–31]. In this study, the potent capabilities of modern computational prediction and bioinformatics were harnessed to search for new inhibitors targeting EGFR.

Marine-based natural compounds play a pivotal role in the creation of innovative pharmaceuticals, particularly those aimed at combating cancer and infectious diseases [32,33]. Several drugs that are currently approved or under clinical trials are sourced from marine, examples being cytarabine, nelarabine, and vidarabine [34]. As a result, there is a significant opportunity to discover fresh candidates for anticancer drugs, specifically wild-type EGFR inhibitors, within the Natural Products Atlas (NPAtlas) database. Furthermore, effective virtual screening across a range of active compounds sourced from different databases, the discovery of potential EGFR inhibitors could lead to novel predictive strategies in the cure of cancer.

## Materials and methods

### Protein and small molecule preparation

The three-dimensional crystal structures of the EGFR proteins (PDB ID: 1M17 for the active form and PDB ID: 1XKK for the inactive form) were retrieved from the RCSB Protein Data Bank (https://www.rcsb.org) and opened in Discovery Studio visualizer tool. Upon examination, it was noted that the structures exhibited disruptions attributable to the absence of certain amino acid residues. To rectify this, the structures underwent repair or reconstruction using the SwissModel web tool (https://swissmodel.expasy.org) regarding self-templates. Subsequently, the models were opened in the Discovery Studio visualizer (DSV), where small molecules such as water, and any additional ligand entries occupying EGFR binding sites were removed.

For virtual screening, ligands were downloaded from different databases including the ZINC database [35], Maybridge database (https://www.thermofisher.com), NPAtlas (Natural Products Atlas) [36], PKIDB (Protein kinase inhibitor database) [37], and Asinex kinase library (https://www.asinex.com/kinase). For the ZINC database trenches with Log values of 2, 3, and 4 and molecular masses ranging from 400–500 g/mol were downloaded. Furthermore, other filters used for ZINC database trenches were structures = 3D, reactivity = anodyne, purchase = in-stock, pH = reference, and charges = -2 to + 2. For NPAtlas only marine-derived compounds were used; for the Maybridge database, both kinase library and hit discover library were used.

### Molecular docking

Virtual screening and molecular docking of compounds are acknowledged as pivotal strategies for advancing the development and delivery of novel drug candidates, that significantly reduces both time and financial investments. The process involved converting the protein files and ligand files into pdbqt format followed by molecular docking using AutoDock Vina [38]. For 1M17, the grid box size in X, Y, and Z coordinates was set at 30 Å × 30 Å × 30 Å, centered at X = 23.00, Y = 0.00, and Z = 56.00, respectively. Similarly, for 1XKK, the grid box size was 30 Å × 30 Å × 30 Å, centered at X = 18.866, Y = 35.252, and Z = 37.650, with a grid spacing of 1.00 Å and exhaustiveness of 8. Discovery Studio Visualizer was utilized to analyze both the three-dimensional and two-dimensional conformations of the ligands within the protein’s active sites.

### Molecular dynamics (MD) simulations

MD simulations were performed on the most energetically favorable configurations derived from the molecular docking investigations using GROMACS (Version 2023.1) [39], with the CHARMM36-jul2022 force field [40] and the TIP3P model [41]. The ligands parameters and topologies were generated through the online CHARMM General Force Field server. To ensure a minimum distance of 2 nm between consecutive images of the 1M17-ATP/Inhibitor and 1XKK-ATP/Inhibitor complexes within a cubic box, the complexes were positioned at least 1 nm away from the box edges, employing periodic boundary conditions. The system’s charge neutrality was maintained by adding one Cl^–^ ion and three Na^+^ ions to the 1M17-Ligand and two Cl^–^ ions to the 1XKK-Ligand complexes. The systems underwent energy minimization until the highest force reached below 10 kJ/mol/nm using the steepest descent algorithm followed by the conjugate gradient protocol. Subsequently, equilibration was performed for 100 ps under isochoric-isothermal (NVT) conditions at 300 K with a time step of 2 fs. Isothermal-isobaric (NPT) equilibration was then conducted at 300 K for 100 ps, utilizing a modified Berendsen thermostat and a time step of 2 fs, while electrostatic and van der Waals interaction cutoffs were set at 1.0 nm. Long-range interactions were computed using the smooth particle mesh Ewald (PME) method [42]. The equilibrated ensembles were subjected to a 100 ns molecular dynamics simulation (production run) with consistent electrostatic and van der Waals cutoffs. The PME method was employed to calculate long-range electrostatic interactions, while a modified Berendsen thermostat and a Parinello-Rahman barostat were used with reference temperature and pressure set at 300 K and 1 bar, respectively.

The average protein structures of apo and NPA008122 bound EGFR proteins were analyzed in ProDy [43] to find the most prominent dynamic region. The nmd files generated were processed in Normal Mode Analysis (NMA) using VMD and were compared. PCA has proven to be an effective strategy for uncovering inherent protein motions and evaluating folding kinetics [44]. Employing the essential dynamics technique, PCA was conducted, and free energy landscapes (FELs) were assessed based on the simulated trajectory generated. PCA, a mathematical method for reducing a multidimensional set of variables to a lower dimension using covariance matrices, was employed. The technique involves eigenvector diagonalization of the covariance matrix, enabling the exploration of conformational selection in both active and inactive EGFR and its interactions with ligands. FELs were constructed utilizing ligands to examine the folding dynamics of EGFR in both apo and complex states. The complexation of EGFR with ligands provided valuable insights into its stability.

### MM-PBSA study

The MM-PBSA package plays a crucial role in conjunction with GROMACS for determining the binding free energy (BE) of ligand-bound complexes. Utilizing the Molecular Mechanic/Poisson-Boltzmann Surface Area (MM-PBSA) approach facilitates the calculation of binding energy [45,46]. Specifically, binding energy calculations were conducted for ATP and Erlotinib, and virtually screened ligands examining their interactions with the active site of the EGFR protein. The binding energies of the complexes were derived from molecular dynamic (MD) simulation trajectories. The representation of the binding energy (${\Delta G}_{binding}$) in the ligand-bound protein complex was determined through the equation:


$${\Delta G}_{binding}=G_{complex}-(G_{protein}+G_{ligand})$$

In this equation, $G_{complex}$ represents the energy of the ligand-bound protein complex, while $G_{protein}$ and $G_{ligand}$ denote the protein and ligand energy in a water-surrounded environment, respectively.

### SwissADME analysis

An analysis using SwissADME (http://www.swissadme.ch) was performed on the top hits to forecast the ADME parameters, pharmacokinetic properties, drug-likeness, and medicinal chemistry suitability of the ligands.

## Results and discussion

### Preparation of the molecules and molecular docking analysis

The active (PDB ID: 1M17) and inactive (PDB ID: 1XKK) structures of EGFR proteins were retrieved from the protein data bank and visualized using the Discovery Studio Visualizer tool. It was observed that the structures exhibited fractures due to the absence of certain amino acid residues. Subsequently, the structures were repaired or reconstructed using the SwissModel online tool with a self-template. Upon completion, the models were imported into DSV to eliminate water molecules and other ligand entries occupying the vacant EGFR binding sites. To initiate virtual screening for potential EGFR kinase inhibitors, essential for combating cancer cell proliferation, a library of approximately 633,000 chemicals was sourced from databases including ZINC, Maybridge, NPAtlas, PKIDB, and Asinex kinase. Molecular docking studies, vital for predicting the precise binding configurations of small molecules serving as ligands at the target site, were conducted using AutoDock Vina software. This method provided insights into receptor-ligand interactions, binding energy, and intermolecular distances between binding residues. The primary objective of this study was to perform virtual screening across various compound libraries to identify potential EGFR kinase inhibitors. Following receptor and small molecule preparation, molecular docking analysis was conducted. The top 15 molecules, selected based on their binding affinity from a library of 633,000 compounds, were optimized. All selected compounds exhibited considerable interactions with the EGFR protein, with binding energy values ranging from -13.8 to -12.2 kcal/mol (refer to Tables 1 and 2).

**Table 1:** Top 15 HITs interacting with the 1M17 protein, with the energies ranging between -13.1 to -12.2 kcal/mol. Chemical structures of ligands are given in S1 Table.

S. No.	Ligand ID	Binding Free Energy (kcal/mol)
**1**	BTB11079	-13.1
**2**	NPA020806	-13.0
**3**	NPA032595	-12.9
**4**	NPA007259	-12.7
**5**	RJC02094	-12.6
**6**	NPA006118	-12.6
**7**	JFD00848	-12.5
**8**	ZINC000017027411	-12.3
**9**	NPA015124	-12.3
**10**	NPA008122	-12.3
**11**	ZINC000170620091	-12.2
**12**	JFD00243	-12.2
**13**	BTB11140	-12.2
**14**	NPA016333	-12.2
**15**	NPA030739	-12.2
**16**	Erlotinib	-7.1
**17**	ATP	-7.6

**Table 2:** Top 15 HITs interacting with the 1XKK protein, with energies ranging between -13.8 to -12.6 kcal/mol. Chemical structures of ligands are given in S2 Table.

S. No.	Ligand ID	Binding Free Energy (kcal/mol)
**1**	BTB13628	-13.8
**2**	BTB13627	-13.5
**3**	NPA032595	-13.3
**4**	BTB11079	-13.2
**5**	JFD00243	-12.9
**6**	NPA015124	-12.9
**7**	NPA027669	-12.9
**8**	MBX048666	-12.7
**9**	NPA007259	-12.7
**10**	NPA030938	-12.7
**11**	ZINC000014241511	-12.7
**12**	ZINC000008299978	-12.6
**13**	ZINC000257243713	-12.6
**14**	ZINC000035482583	-12.6
**15**	ZINC000033088664	-12.6

Based on binding energies, 15 compounds were prioritized for each protein and their interactions with the proteins were analyzed. These 15 compounds interacted with 1M17 and 1XKK with strong affinities, i.e., -13.8 kcal/mol and -12.2 kcal/mol, respectively sharing the same binding pockets (S2 and S3 Figs).

### Validation of docking

**Fig 1:**
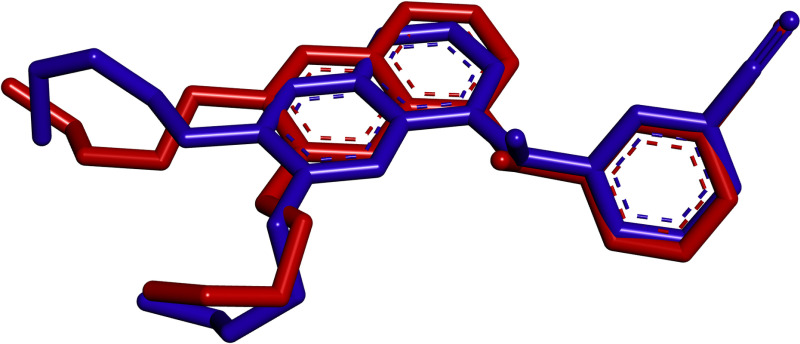
Validating the docking process involves re-docking erlotinib to the EGFR protein (PDB ID: 1M17). The crystal conformation of the ligand is depicted using red sticks, while the docked pose is illustrated with blue sticks.

The erlotinib co-crystallized form was retrieved from the EGFR protein PDB file. This extracted ligand underwent redocking into the protein using the same parameters and road map to verify the consistency and reproducibility of the docking outcomes. The RMSD value was calculated using all atoms in the DSV tool. The RMSD value for the docked ligand compared to the co-crystallized ligand was 1.21 Å. The docked erlotinib and the co-crystallized structure of erlotinib exhibited significant overlap, as depicted in Fig 1. These findings strongly support the conclusion that the docking experiment generated accurate docking poses, thereby validating the results.

### Molecular interactions of ligands

The top 15 compounds in both studies formed key interactions with active site residues of 1M17 and 1XKK including hydrogen bonds and other hydrophobic interactions. The residues of 1M17 involved in hydrogen bond formation with ligands are K721, T766, Q767, M769, C773, T830, and D831. The residues of 1XKK involved in hydrogen bond formation with ligands are L694, A698, K721, Q767, M769, T830, and D831. Other hydrophobic interactions are summarized in S3 and S4 Tables. The docking results indicated that hydrocarbon ligands such as BTB11079 and BTB11140 did not form any hydrogen bond interactions. The stability of their complexes was primarily due to hydrophobic interactions. For protein 1M17, it was observed that most of the ligands formed hydrogen bonds with the side chain residues M769, K721, T766, and T830. For the protein 1XKK, it was observed that the complexes were more stable compared to those formed by the protein 1M17. Although the number of hydrogen bond interactions in the 1XKK complexes was smaller compared to the 1M17 complexes, the number of residues involved in hydrophobic interactions was greater in the 1XKK complexes. Therefore, it can be inferred that the inactive form of EGFR is involved in forming a greater number of hydrophobic interactions, which subsequently results in more stable complexes.

### Molecular dynamic simulations

While conducting molecular dynamics (MD) simulations for the receptor and control complex, a thorough analysis of various parameters was conducted in order to comprehensively evaluate the system’s behavior. The parameters considered included the root mean square deviation (RMSD), root mean square fluctuation (RMSF), principal component analysis (PCA), and MM-PBSA. The molecular dynamics (MD) simulations were conducted for all 15 HITs obtained from the molecular docking study against 1M17 and 1XKK. The MD results were visualized in VMD to assess the stabilities of the complexes. Upon trajectory analysis, it was observed that certain complexes exhibited instability, with ligands leaving the active site of the proteins. The ligand BTB11079, which formed the most stable complex with 1M17 in the docking study, was observed to leave the active site at approximately 30 ns and then re-interact with the surface of the protein at 44 ns during the MD simulation. This behavior was also noted for the BTB11140-1M17 complex. BTB11079, a hydrocarbon ligand, produced a stable result in molecular docking against 1XKK. Analysis of the 1XKK-BTB11079 complex trajectory in VMD revealed that it formed a more stable complex compared to those formed by 1M17 and the hydrocarbon ligands. These results are consistent with the pattern observed in the docking studies, indicating that the inactive form of EGFR interacts more effectively with the ligands through hydrophobic interactions. To narrow down the number of ligands targeting EGFR proteins, the top five ligands based on their binding energies were calculated using MM-PBSA and by analyzing their trajectories were filtered. Ligands that exhibited the most negative binding energies and remained in the active site throughout the MD simulation were selected for further analysis. RMSD graphs of ligands fitting in both 1M17 and 1XKK are presented in S4 and S5 Figs, respectively. Those complexes that displayed disruption were removed from the analysis. The free binding energies calculated using MM-PBSA for all 15 HITs against 1M17 and 1XKK are given in S5 Table. As illustrated in Table 4, the five complexes with the lowest binding energies were further investigated for both 1M17 and 1XKK.

### Global stability indices – RMSD/RMSF

**Fig 2:**
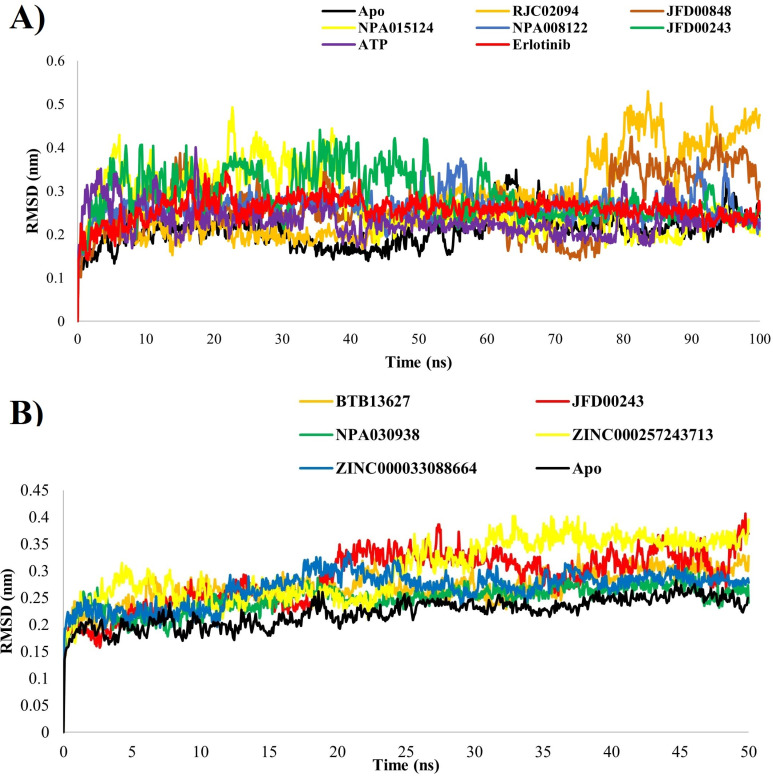
Graphs depicting the Root Mean Square Deviation (RMSD) for complexes (A = 1M17, B = 1XKK) in both ligand-bound and ligand-unbound states.

To gauge the stability and fluctuations within the system, the RMSD was specifically focused, which quantifies the variation in the protein structure backbone during transitions between different conformations. The RMSD provides a quantitative measure of the ligand’s stability concerning the protein and its binding pocket. The observed changes at the end of the simulation were predominantly localized around a thermal average structure, indicating equilibration. Notably, the protein associated with ligands underwent observable conformational changes throughout the simulation, as evidenced by the convergence of the simulation and the stabilization of RMSD values, which persisted until the 100 ns mark (Fig 2A).

Table 3 reveals that, in 1M17 systems, there is a consistent pattern with values hovering around 0.26 nm, except for RJC02094 and JFD00848, which exhibit slightly elevated amplitude of fluctuation in the latter portion of the simulations. When transitioning from the apo to the bound state of the inactive EGFR protein, there was a discernible uptick in fluctuation amplitude, indicating heightened flexibility upon substrate/inhibitor binding within the active pocket (Fig 2B). In general, the trajectories remained stable throughout the time explored in both EGFR active and EGFR inactive protein systems.

**Table 3:** Average Root Mean Square Deviation values of ligand bound and unbound proteins.

System	Average RMSD
1M17-Apo-Protein	0.21
1M17-ATP-Complex	0.23
1M17-JFD00243-Complex	0.29
1M17-NPA015124-Complex	0.27
1M17-RJC02094-Complex	0.28
1M17-NPA008122-Complex	0.26
1M17-JFD00848-Complex	0.27
1M17-Erlotinib-Complex	0.26
1XKK-Apo-Protein	0.22
1XKK-BTB13627-Complex	0.26
1XKK-JFD00243-Complex	0.28
1XKK-NPA030938-Complex	0.24
1XKK-ZINC000257243713-Complex	0.30
1XKK-ZINC000033088664-Complex	0.26

**Fig 3:**
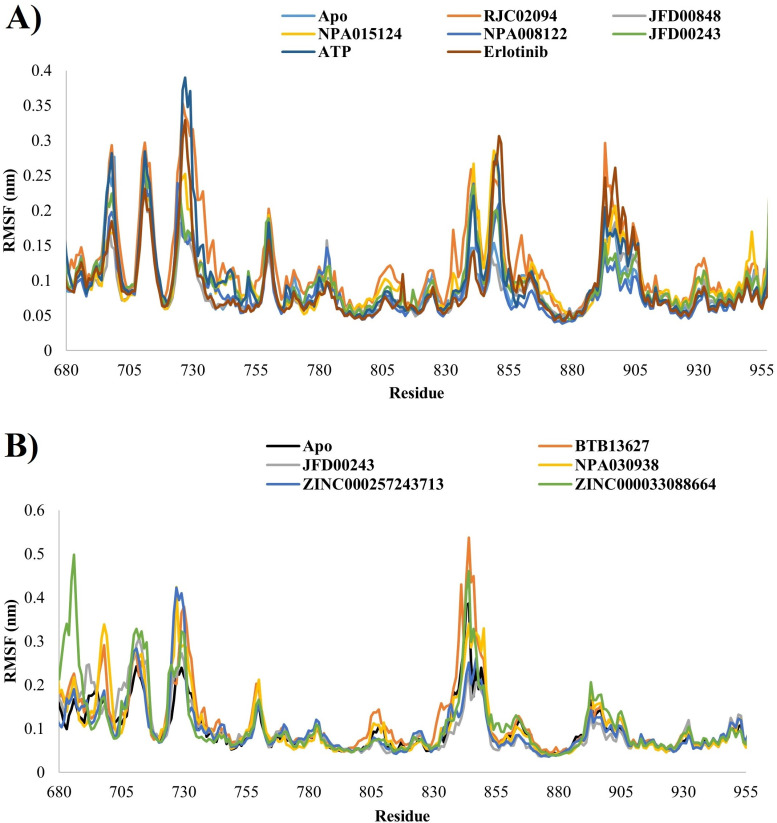
Graphs depicting the Root Mean Square Fluctuation (RMSF) for complexes (A = 1M17 and B = 1XKK) in both ligand-bound and ligand-unbound states.

To further discern and analyze localized alterations in the protein chain, Residual Mean Square Fluctuation (RMSF) was employed. The RMSF analysis revealed minimal variations, indicating a stable interaction between the protein and ligands. Fig 3A and 3B illustrate the root mean square fluctuation of the protein, displaying subtle fluctuations that contributed to the formation of a complex with ligands, albeit in a manner challenging to discern in EGFR active/inactive proteins.

**Fig 4:**
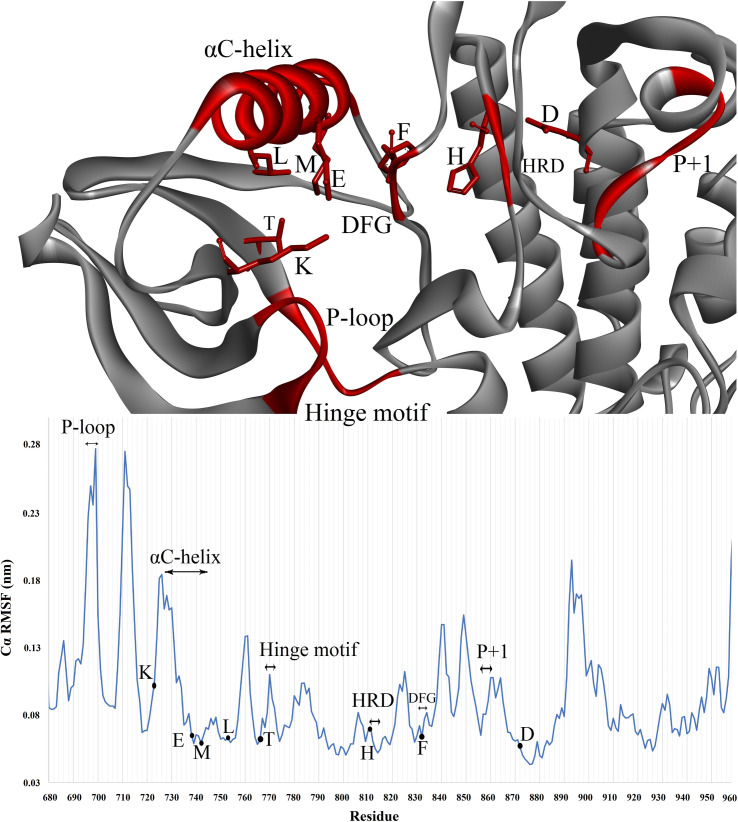
Important residues, loops, and motifs in human EGFR protein and Root Mean Square Fluctuation (RMSF) graph of apo protein.

Comparing the root mean square fluctuations (RMSF) obtained from molecular dynamics (MD) simulations of both the active and inactive states of EGFR proteins in their respective bound and unbound states elucidates a distinctively heightened RMSF profile across the entirety of the EGFR active conformation, whereas subdued fluctuations characterize the inactive EGFR. Fig 4 illustrates functionally significant residues of the EGFR protein kinase, accompanied by a graph displaying RMSF. The primary regulatory components in the kinase catalytic domain of EGFR contain the αC-helix and the DFG motif. The structural interconnection of the regulatory αC-helix and the DFG motif has long been acknowledged as pivotal in managing a dynamic balance between key functional states, including an inactive state (DFG-out/αC-helix-in) and an active state (DFG-in/αC-helix-in). Additionally, crucial residues such as K721 assist in anchoring ATP α- and β-phosphates, while E738 establishes electrostatic interactions with K721. T766 serves as a gatekeeper residue, and D813 acts as a catalytic base. The regulatory spine (R-spine) residues (M742, L753, H811, F832, and D872) play a significant role in governing protein kinase regulation.

### Ligands and active site residues distance analysis

**Fig 5:**
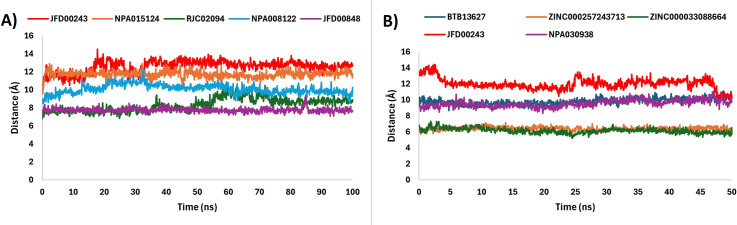
The change in distance between the ligands and side chain residues of EGFR proteins. **A)** 1M17-Ligand complexes, and **B)** 1XKK-Ligand complexes, respectively.

To investigate the structural shifts in the binding pockets and the behavior of ligands within the active sites of 1M17 and 1XKK we calculated the distances between the ligands and side chain residues (E687 to R724, P729 to S744, V750 to P770, and A816 to K836) of the active sites. As shown in Fig 5A, except for RJC02094 no major changes were observed in the distances between the ligands and active site residues of the 1M17 protein. It is evident that the distance between RJC02094 and active site residues of 1M17 equilibrated at same distance up to 55 ns; afterward, the distance increased from 8 Å to 10 Å for rest of the simulation time. On the other hand, for 1XKK-ligand complexes the distance between the ligands and the residues of the active site maintained same distances throughout the simulations. As shown in Fig 5B, JFD00243 showed small fluctuations in distances both at the start and at the end of the simulation. Distance plots indicate that the selected ligands maintained specific positions throughout the simulation. The interaction between the best ligands and the EGFR proteins is significantly influenced by these alterations in spatial distances.

### ANM analysis for mechanical stiffness

**Fig 6:**
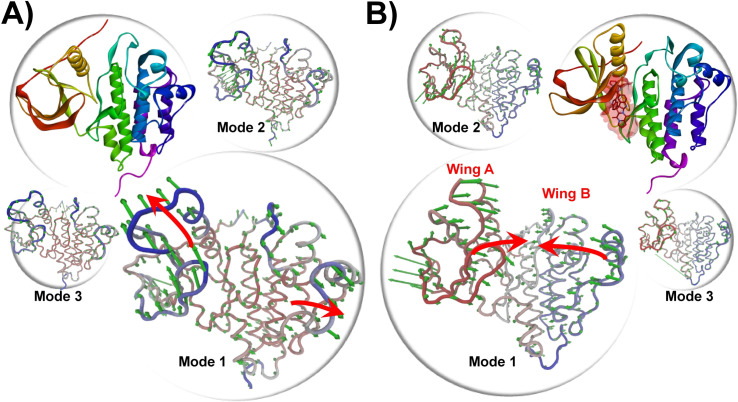
The Anisotropic Network Model (ANM analysis) of the apo EGFR protein (A) and NPA008122 bound EGFR protein (B). The backbone is shown in tube form and the green arrows indicate the degree of elasticity.

Molecular dynamics (MD) simulations of the Epidermal Growth Factor Receptor (EGFR) revealed distinct conformational differences between its bound and free states, as evidenced by RMSD and RMSF analyses. These studies showed that in the bound state, EGFR undergoes notable inward movements of the wing A and wing B regions (Fig 6), facilitating the closure of a protein loop between these wings. In contrast, the free state displays outward movements of the wings, resulting in a more open loop conformation. Anisotropic Network Model (ANM) analysis confirmed these findings, with Mode 1 showing pronounced inward and outward flap movements, and Modes 2 and 3 capturing less pronounced but complementary dynamics.

These observations suggest that ligand binding induces a conformational shift in EGFR, stabilizing a closed state that enhances the receptor’s functional stability by closing off the cleft between the wings. This structural adjustment is crucial for the receptor’s activity and interactions with signaling molecules. Conversely, the more flexible, open conformation observed in the free state may be necessary for effective ligand binding and regulatory processes. Overall, the differential dynamics highlighted by RMSD, RMSF, and ANM analyses underscore the significant structural rearrangements upon ligand binding and emphasize the role of conformational flexibility in EGFR function and regulation.

### Buried solvent-accessible surface area analysis

Buried solvent-accessible surface area (B-SASA) is the surface area which is inaccessible to solvent where the ligand and protein interacts. B-SASA is used to quantify the interactions between the ligand and the protein. B-SASA for the 1M17-ligand complexes was calculated using the expression:


$$B\text{-}SASA=0.5\left(SASA_{Ligand}+SASA_{Protein}-SASA_{Complex}\right)$$

**Fig 7:**
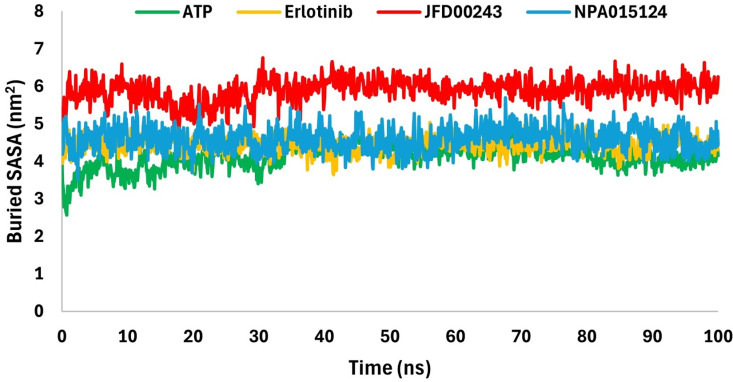
Buried solvent-accessible surface area (B-SASA) values for 1M17-ATP, 1M17-Erlotinib, 1M17-JFD00243, and 1M17-NPA015124 complexes.

The mean values for B-SASA calculated for 1M17-ATP, 1M17-Erlotinib, 1M17-JFD00243, and 1M17-NPA015124 complexes are 4.12 nm^2^, 4.44 nm^2^, 5.90 nm^2^, and 4.60 nm^2^, respectively (Fig 7). It is seen that JFD00243 and NPA015124 values are greater than the ATP and Erlotinib values. It can be concluded from B-SASA values that JFD00243 interacts more strongly with the protein.

### MM-PBSA analysis

The MM-PBSA analysis reveals distinct energy components contributing to the overall binding energy of protein-ligand complexes (Table 4). ATP exhibits the most favorable binding interaction with a binding energy of -66.67 kcal/mol, indicating strong attractive forces, prominent electrostatic energy, and a hydrophobic environment within the active site. Among the inhibitors, JFD00243 displays a substantial negative binding energy of -32.16 kcal/mol, emphasizing hydrophobic interactions and potential solvent exposure. NPA015124 shows a binding energy of -22.67 kcal/mol, highlighting significant contributions from both van der Waals and electrostatic energies, with a balance between solvent exposure and hydrophobic interactions. Similarly, RJC02094, with a binding energy of -22.21 kcal/mol, demonstrates favorable binding characterized by notable van der Waals and electrostatic energies, suggesting a balanced solvent exposure and hydrophobic environment. NPA008122 interacts with active pocket residues, resulting in a binding score of -20.85 kcal/mol, indicating a balance between solvent exposure and hydrophobic interactions. In summary, negative binding energies across all selected complexes indicate favorable binding interactions. Van der Waals and electrostatic energies play pivotal roles, while polar solvation and SASA energies provide insights into solvent accessibility and hydrophobic interactions. The ranking of compounds based on descending binding energies places ATP, JFD00243, NPA015124, RJC02094, NPA008122, JFD00848, and Erlotinib in order of increasing binding affinity for EGFR active protein. Contrary data represents the MM-PBSA simulations for the inactive state of EGFR. The EGFR inactive-BTB13627 shows a binding energy of -32.52 kcal/mol and the complex exhibits the highest negative binding energy among the provided results, suggesting a favorable binding affinity between EGFR in its inactive state and the ligand BTB13627. The negative value indicates that the interaction is energetically favorable. The contributions to the binding energy include significant favorable contributions from van der Waals interactions (-58.65) and polar solvation energy (40.61). The EGFR inactive-ZINC000257243713 binds with an energy of -28.25 kcal/mol, while this complex has a slightly less negative binding energy compared to the previous one, it still indicates a favorable binding interaction. Contributions from van der Waals interactions (-54.84) and polar solvation energy (43.74) are notable in this case. Similarly, EGFR inactive-ZINC000033088664 with a binding Energy of -27.61 supported primarily by van der Waal interactions also appears as a good inhibitor in terms of its binding in the active pocket. These results suggest that BTB13627 has the strongest binding affinity for EGFR in its inactive state among the ligands investigated. Based on the MMPSA results, it is evident that the complexes formed by the inactive form of EGFR are more stabilized by van der Waals interactions compared to those formed by the active form of the EGFR protein. This finding is also supported by the greater number of hydrophobic interactions observed in the docking study for the inactive EGFR protein. The per-residue energy decomposition analysis from MMPBSA, illustrating the contribution of individual residues to the total binding energy for all complexes, is provided in Supporting Information (S6 Fig).

**Table 4:** Binding free energies (in kcal/mol) for the 1M17-Ligand and 1XKK-Ligand complexes calculated using MM-PBSA.

Complex	Binding Energy kcal/mol	van der Waal Energy kcal/mol	Electrostatic Energy kcal/mol	Polar solvation Energy kcal/mol	SASA Energy kcal/mol
1M17-ATP	-66.67	-23.25	-254.82	215.79	-4.39
1M17-JFD00243	-32.16	-59.34	-2.62	36	-6.2
1M17-NPA015124	-22.67	-41.79	-24.24	48.17	-4.8
1M17-RJC02094	-22.21	-46.34	-13.01	41.97	-4.82
1M17-NPA008122	-20.85	-48.44	-3.31	36.37	-5.46
1M17-JFD00848	-19.43	-50.99	-9.62	46.311	-5.11
1M17-Erlotinib	-11.92	-40.63	-10.6	44.11	-4.79
1XKK-BTB13627	-32.52	-58.65	-8.37	40.61	-6.1
1XKK-ZINC000257243713	-28.25	-54.84	-11.58	43.74	-5.56
1XKK-ZINC000033088664	-27.61	-55.69	-10.01	43.35	-5.25
1XKK-JFD00243	-25.93	-53.33	-6.79	40.06	-5.86
1XKK-NPA030938	-24.42	-55.14	-9.28	45.75	-5.74

### Principal component analysis

Principal Component Analysis (PCA) is a statistical method widely employed in molecular dynamics simulations to reduce the dimensionality of complex data sets while retaining essential structural information. In the context of protein dynamics, PCA helps identify the principal modes of motion and highlights the most significant collective motions occurring within the system. When applied to MD trajectories of proteins, PCA analyzes the covariance matrix of atomic fluctuations, extracting principal components that represent the major modes of motion. These principal components can then be used to project the trajectory onto a lower-dimensional space, such as a 2D phase space. The resulting visualization helps to elucidate the dominant structural variations and dynamic behaviors of the protein.

**Fig 8:**
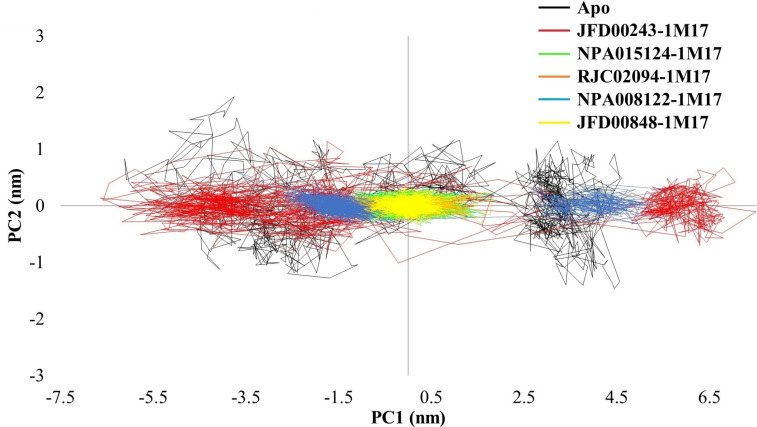
Principal Component Analysis (PCA) of unbound Apo-1M17 and ligand-bound 1M17 proteins.

In the case of Apo EGFR (active), the broader conformational space observed in the phase space suggests increased flexibility and diverse structural transitions, characteristic of an unbound protein exploring various conformations. Conversely, the more compact phase space observed in EGFR bound to ATP and ligands including erlotinib indicates a constrained and specific set of motions. The overlapping region with the apoprotein suggests that certain motions remain conserved in the ligand-bound states but with a more defined and restricted conformational space (Fig 8).

**Fig 9:**
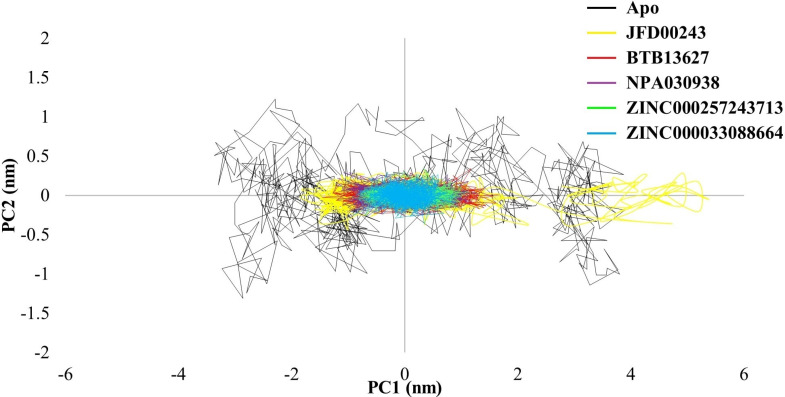
Principal Component Analysis (PCA) of unbound Apo-1XKK and ligand-bound 1XKK proteins.

In the case of Apo EGFR (inactive), a broader conformational space suggests increased flexibility and diverse structural transitions. Conversely, inactive EGFR in its bound state exhibits a more compact phase space, indicating constrained and specific motions. However, there is an overlap with the apo protein, suggesting that some motions remain conserved in the ligand-bound states but with a more defined conformational space (Fig 9).

The free energy landscape (FEL) comparison between a protein-bound complex and a free protein typically involves analyzing the distribution of conformations and associated energies in a multidimensional space. In the context of a molecular dynamics simulation, the free energy landscape illustrates the probability of finding the system in different conformational states. If the bound protein complex exhibits more blue regions in the free energy landscape compared to the free protein, it suggests that the complex has a more stable and energetically favorable conformational space. Blue regions often represent lower free energy states, indicating more thermodynamically stable conformations. This phenomenon could be attributed to the interactions between the protein and its binding partner in the complex. When the protein is bound to its ligand or partner, specific interactions such as hydrogen bonds, hydrophobic interactions, and electrostatic interactions contribute to the stabilization of certain conformations. These interactions may lead to a more confined and favorable conformational space for the protein within the complex, resulting in a larger blue region on the free energy landscape compared to the free protein. The analysis of the free energy landscape provides a comprehensive understanding of the energetically favorable states and transitions within a protein-ligand system. Free energy landscapes are often generated from molecular dynamics (MD) simulations and depict the distribution of conformations in a multidimensional space, with lower free energy regions representing more stable states.

In the context of the mentioned PCA analysis for Apo 1M17, 1M17-ATP, 1M17-Erlotinib, 1M17-inhibitors complexes (Fig 8), and 1XKK-inhibitors complexes (Fig 9), the resulting two-dimensional projections in phase space offer insights into the systems free energy landscape. The distribution of conformations and their relative energies can be inferred from the density of points in the phase space plot.

The comparison of free energy landscapes (FEL) between the Apo protein, ATP-bound (EGFR-ATP), Erlotinib-bound (EGFR-Erlotinib), and inhibitor-bound (EGFR-inhibitors,) proteins sheds light on their structural dynamics and stability. For Apo 1M17, the broader conformational space observed in the phase suggests a more diverse range of protein conformations with varying free energy levels. Peaks and valleys in the landscape represent distinct energy minima and maxima, respectively, corresponding to different structural states explored by the unbound protein. The minimum energy conformation M1 extracted from the FEL of unbound 1M17 sampled a secondary structure content of 36.9% helix, 19.5% sheet, 13.3% turn, and 30.4% coil as listed in Table 5.

**Table 5:** The secondary structure statistics of the minimum-energy basin conformations extracted from the FELs of 1M17 and 1XKK in the absence and presence of screened compounds.

Protein/Protein-ligand complex	Conformation	Secondary structure component (%)			
		Coil	Sheet	Turn	Helix
1M17	M1	30.4	19.5	13.3	36.9
1M17-ATP	M2	33.8	20.1	12.3	32.4
1M17-Erlotinib	M3	28.3	19.8	15.7	36.2
1M17-RJC02094	M4	32.4	18.8	13.7	35.2
	M4*	31.1	20.1	15.0	33.8
1M17-JFD00848	M5	29.0	19.1	17.1	34.8
1M17- NPA015124	M6	30.0	20.1	14.3	35.5
1M17-NPA008122	M7	35.5	18.1	10.9	35.5
1M17-JFD00243	M8	35.2	16.7	11.6	36.5
1XKK	X1	38.0	11.0	16.7	34.3
1XKK-BTB13627	X2	34.7	13.3	17.7	34.3
1XKK-JFD00243	X3	38.3	14.3	11.0	36.3
1XKK-NPA030938	X4	34.7	12.3	16.3	36.7
	X4*	37.3	11.0	15.0	36.7
1XKK-ZINC000257243713	X5	34.3	12.3	17.7	35.7
1XKK-ZINC000033088664	X6	30.0	15.3	15.0	38.3

1M17-ATP and 1M17-Erlotinib with more compact phase space in these ligand-bound complexes indicate a constrained set of motions and a narrower range of explored conformations. This compactness implies a more stable and defined free energy landscape, with the ligands influencing the proteins dynamics and stabilizing specific conformations. The secondary structure compositions—32.4% helix, 20.1% sheet, 12.3% turn, and 33.8% coil for M2 conformation of ATP, and 36.2% helix, 19.8% sheet, 15.7% turn, and 28.3% coil for M3 conformation of Erlotinib—suggest a well-structured and stable environment (Table 5).

The Free Energy Landscape (FEL) plots of 1M17 under different conditions reveal distinct conformational dynamics. In the absence of ATP, 1M17 exhibits limited conformational flexibility, reflected by a small basin and narrow space explored. However, in the presence of ATP, the protein explores a wide conformational landscape with multiple low-energy structures, indicating significant conformational changes induced by ATP binding. Similarly, bound states with inhibitors like Erlotinib, RJC02094, JFD00848, NPA015124, and JFD00243 also lead to wider energy landscapes, suggesting substantial conformational flexibility influenced by these compounds. Interestingly, NPA008122 binding results in a very narrow FEL with two small clusters, indicating a highly constrained conformational state compared to the other compounds. In conclusion, different compounds interact with 1M17 in distinct ways, inducing varying degrees of conformational changes and flexibility, with ATP and certain inhibitors promoting wide conformational landscapes, while NPA008122 binding restricts conformational flexibility to a greater extent.

**Fig 10:**
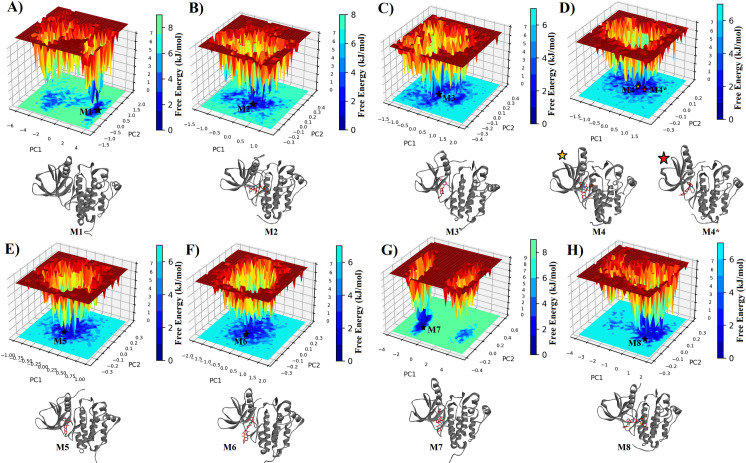
The FEL plots of 1M17 in the absence (A) and presence of ATP (B), Erlotinib (C), RJC02094 (D), JFD00848 (E), NPA015124 (F), NPA008122 (G), and JFD00243 (H) compounds, respectively. 3-D protein structures individually represent the lowest energy conformation(s) retrieved from FEL.

The overlapping regions in the phase space between Apo 1M17 and ligand-bound states suggest that certain protein motions are conserved across different states. These shared regions may correspond to stable conformations that persist with or without ligand binding, influencing the overall free energy landscape (Fig 10). The M4 and M4^*^ conformations of RJC02094, an inhibitor-bound protein, explore a large conformational space with M4 = 35.2% helix, 18.8% sheet, 13.7% turn, and 32.4% coil and M4^*^ = 33.8% helix, 20.1% sheet, 15.0% turn, and 31.2% coil. All the inhibitors showed significant structural flexibility, except for JFD00243, which explores a smaller portion of the free energy landscape, suggesting a more constrained structure.

**Fig 11:**
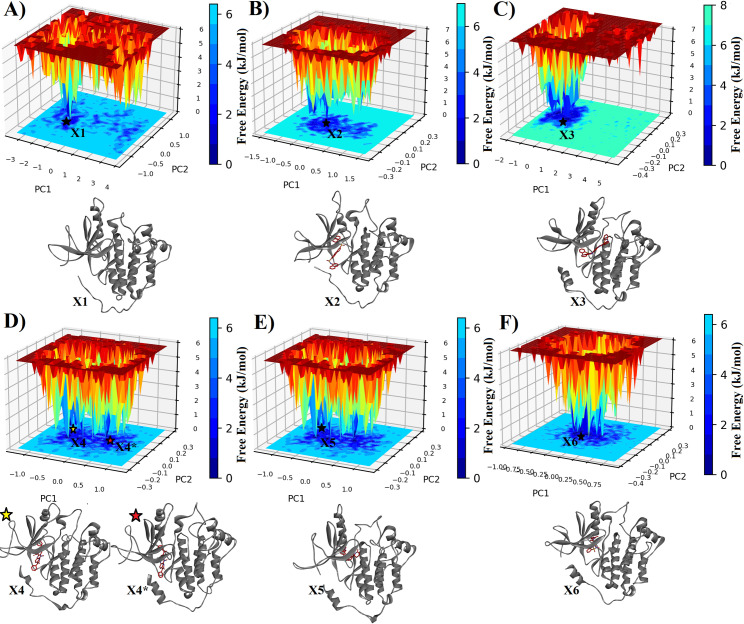
The FEL plots of 1XKK in the absence (A) and presence of BTB13627 (B), JFD00243 (C), NPA030938 (D), ZINC000257243713 (E), and ZINC000033088664 (F) compounds, respectively. 3-D protein structures individually represent the lowest energy conformation(s) retrieved from FEL.

The FEL plots of 1XKK depict distinct conformational dynamics under different conditions. In the absence of an inhibitor, the protein exhibits a dispersed FEL with multiple small clusters, indicating a range of conformational states (Fig 11). However, in the presence of BTB13627, a more confined, yet definite, small cluster is observed, suggesting limited structural variations despite experiencing multiple conformations. A similar trend is observed with the inhibitor JFD00243, where the presence of the inhibitor leads to a confined FEL with limited structural variations. Conversely, for the compounds NPA030938, ZINC000257243713, and ZINC000033088664, a wide conformational space is explored by the bound protein system, with multiple blue regions indicating significant structural flexibility and variation. These observations underscore the diverse effects of different compounds on the conformational landscape of 1XKK, with some inducing confined clusters with limited structural variations and others promoting wide conformational spaces with greater flexibility.

### SwissADME analysis

The bioavailability of the molecules screened virtually, along with the most potent inhibitors in the dataset, was assessed to ascertain the viability of proposed drug candidates and their synthetic accessibility. Specific pharmacokinetic properties were estimated accordingly. As depicted in the S6 Table, except for BTB13627 and JFD00243, most of the screened compounds exhibit moderate solubility in water, and gastrointestinal (GI) absorption is notably high for ZINC000033088664, ZINC000257243713, NPA008122, JFD00848, and RJC02094. These findings suggest the potential of these drugs for oral formulation. Additionally, ZINC000033088664, ZINC000257243713, JFD00848, and RJC02094 demonstrate blood-brain barrier (BBB) permeation capabilities. Evaluation of drug-likeness indicates that ZINC000033088664, ZINC000257243713, NPA030938, NPA008122, JFD00848, and RJC02094 comply with Lipinski’s rule.

## Conclusions

The current computational investigation focused on identifying small molecule inhibitors targeting EGFR which is a pivotal protein in cancer progression. Different compound libraries were screened by integrating molecular docking and dynamics simulations, optimizing top candidates based on binding affinity. Validation through erlotinib redocking underscored the reliability of docking approach used in this study. Molecular dynamics simulations unveiled the dynamic behaviors and stability of EGFR-ligand complexes, emphasizing the significance of specific interactions in modulating binding affinities. Notably, the simulations revealed key protein-ligand interactions that are critical for potential inhibition mechanisms. The ANM analysis of EGFR reveals that the significant inward and outward flap movements observed in Mode 1 are complemented by less pronounced but still relevant motions in Modes 2 and 3. These findings highlight the complexity of the protein’s conformational dynamics and underscore the role of lower-frequency modes in supporting the functional flexibility and stability of EGFR in both its bound and free states. The integration of ANM data with RMSD and RMSF analyses provides a comprehensive view of how ligand binding induces specific structural rearrangements and affects the overall dynamics of the receptor.

The results of current study identify five lead anticancer inhibitors that target both active and inactive EGFR proteins. The inhibitors JFD00243, NPA015124, RJC02094, NPA008122, and JFD00848 were found to be effective against active EGFR, while BTB13627, ZINC000257243713, ZINC000033088664, JFD00243, and NPA030938 were effective against inactive EGFR. Notably, JFD00243 was identified as a common inhibitor for both active and inactive EGFR proteins. These inhibitors can be further assessed using experimental and clinical approaches to improve future cancer management.

## Supporting information

S1 Fig Different EGFR inhibitors used for the treatment of Lung cancer. (DOCX)

S1 Table Chemical Structures of the top 15 HITs docked in 1M17 protein. (DOCX)

S2 Table Chemical Structures of the top 15 Hits docked in 1XKK protein. (DOCX)

S3 Table Summary of 1M17 residues interacting with the ligands. (DOCX)

S4 Table Summary of 1XKK residues interacting with the ligands. (DOCX)

S2 Fig Two-dimensional interactions of top 15 compounds, ATP, and Erlotinib with the active site of active EGFR protein (PDB ID: 1M17). (DOCX)

S3 Fig Two-dimensional interactions of top 15 compounds with the active site of inactive EGFR protein (PDB ID: 1XKK). (DOCX)

S4 Fig The RMSD graphs for the ligands fit on 1M17 protein. (DOCX)

S5 Fig The RMSD graphs for the ligands fit on 1XKK protein. (DOCX)

S5 Table Binding free energies (in kcal/mol) for the 1M17-Ligand and 1XKK-Ligand complexes calculated using MM/PBSA. (DOCX)

S6 Fig Per-residue energy decomposition analysis from MMPBSA showing the contribution of individual residues to the total binding energy. (DOCX)

S6 Table Pharmacokinetic profile of screened compounds. (DOCX)
