# Supplementary material for: Unraveling potential EGFR kinase inhibitors: Computational screening, molecular dynamics insights, and MMPBSA analysis for targeted cancer therapy development
Source: PLoS One. 2025 May 9;20(5):e0321500. doi: 10.1371/journal.pone.0321500 (PMC12064201; doi:10.1371/journal.pone.0321500)
Supplement: S1 Table — (DOCX) [file pone.0321500.s002.docx]

**S1 Table.** Chemical Structures of the top 15 HITs docked in 1M17 protein

| **S. No.** | **Ligand ID** | **Structure** |
| --- | --- | --- |
| **1** | BTB11079 |  |
| **2** | NPA020806 |  |
| **3** | NPA032595 |  |
| **4** | NPA007259 |  |
| **5** | RJC02094 |  |
| **6** | NPA006118 |  |
| **7** | JFD00848 |  |
| **8** | ZINC000017027411 |  |
| **9** | NPA015124 |  |
| **10** | NPA008122 |  |
| **11** | ZINC000170620091 |  |
| **12** | JFD00243 |  |
| **13** | BTB11140 |  |
| **14** | NPA016333 |  |
| **15** | NPA030739 |  |
| **16** | Erlotinib |  |
| **17** | ATP |  |
