# Supplementary material for: Unraveling potential EGFR kinase inhibitors: Computational screening, molecular dynamics insights, and MMPBSA analysis for targeted cancer therapy development
Source: PLoS One. 2025 May 9;20(5):e0321500. doi: 10.1371/journal.pone.0321500 (PMC12064201; doi:10.1371/journal.pone.0321500)
Supplement: S2 Table — (DOCX) [file pone.0321500.s003.docx]

**S2 Table.** Chemical Structures of the top 15 Hits docked in 1XKK protein

| **S. No.** | **Ligand ID** | **Structure** |
| --- | --- | --- |
| **1** | BTB13628 |  |
| **2** | BTB13627 |  |
| **3** | NPA032595 |  |
| **4** | BTB11079 |  |
| **5** | JFD00243 |  |
| **6** | NPA015124 |  |
| **7** | NPA027669 |  |
| **8** | MBX048666 |  |
| **9** | NPA007259 |  |
| **10** | NPA030938 |  |
| **11** | ZINC000014241511 |  |
| **12** | ZINC000008299978 |  |
| **13** | ZINC000257243713 |  |
| **14** | ZINC000035482583 |  |
| **15** | ZINC000033088664 |  |
