# Supplementary material for: Unraveling potential EGFR kinase inhibitors: Computational screening, molecular dynamics insights, and MMPBSA analysis for targeted cancer therapy development
Source: PLoS One. 2025 May 9;20(5):e0321500. doi: 10.1371/journal.pone.0321500 (PMC12064201; doi:10.1371/journal.pone.0321500)
Supplement: S3 Table — (DOCX) [file pone.0321500.s004.docx]

**S3 Table.** Summary of 1M17 residues interacting with the ligands

| **S. No.** | **Ligand ID** | **H-bond Interaction** | | | | **Hydrophobic Interaction** |
| --- | --- | --- | --- | --- | --- | --- |
|  |  | **Residue** | **Distance (Å)** | | **Angle** |  |
| **1** | ATP | Q767 | 3.26 | | 10.29 | F669, L694, A698, A719, K721, L768, R817, L820, D831 |
|  |  | M769 | 3.26 | | 156.51 |  |
|  |  | C773 | 3.35 | | 124.08 |  |
| **2** | Erlotinib | M769 | 2.70 | | 169.51 | L694, A719, K721, E738, L764, T766, Q767, L768, P770, F771, G772, L820, T830, D831 |
| **3** | BTB11079 | - | | | - | L694, F669, V702, A719, K721, M769, C773, R817, L820, D831 |
| **4** | NPA020806 | K721 | 2.91 | | 141.57 | A698, F669, V702, A719, M769, G772, L820, D831 |
| **5** | NPA032595 | K721 | 3.33 | | 133.07 | L694, V702, A719, E738, L764, L768, P770, G772, G780, L820, D831 |
|  |  | T766 | 2.81, 2.92 | | 102.22, 49.99 |  |
|  |  | Q767 | 2.88 | | 68.35 |  |
|  |  | M769 | 2.76 | | 69.46 |  |
| **6** | NPA007259 | K721 | 3.31 | | 133.63 | L694, V702, A719, E738, L764, L768, P770, G772, G780, L820, D831 |
|  |  | T766 | 2.83, 2.94 | | 94.96, 48.11 |  |
|  |  | Q767 | 2.89 | | 57.02 |  |
|  |  | M769 | 2.76 | | 139.72 |  |
| **7** | RJC02094 | M769 | 3.03 | | 151.91 | L694, F669, V702, A719, L764, T766, L768, L820, D831 |
|  |  | K721 | 3.29 | | 143.63 |  |
| **8** | NPA006118 | M769 | 3.31 | | 162.69 | L694, F669, V702, A719, K721 E738, M742, L764, T766, G772, C773, L820, D831 |
|  |  | T830 | 2.97 | | 52.51 |  |
| **9** | JFD00848 | M769 | 3.05 | | 154.52 | L694, F669, V702, A719, L764, T766, L768, P770, L820, D831 |
|  |  | K721 | 3.28 | | 143.03 |  |
| **10** | ZINC000017027411 | K721 | 2.97 | | 101.81 | L694, F669, V702, A719, M742, T766, M769, L820, D831 |
|  |  | T830 | 2.85 | | 25.39 |  |
| **11** | NPA015124 | K721 | 3.32 | | 138.17 | L694, V702, A719, E738, L764, L768, P770, G772, G780, L820, T830, D831 |
|  |  | T766 | 2.90, 2.91 | | 88.95, 109.44 |  |
|  |  | Q767 | 2.98 | | 72.55 |  |
|  |  | M769 | 2.80 | | 142.83 |  |
| **12** | NPA008122 | C773 | 3.13 | | 134.16 | L694, G695, V702, A719, K721, E738, M742, L764, T766, M769, P770, F771, G772, D776, L820, T830 |
|  |  | D831 | 3.28 | | 131.10 |  |
| **13** | ZINC000170620091 | T766 | 2.81 | 53.56 | | F669, V702, A719, K721, M742, M769, L820, D831 |
|  |  | T830 | 2.77 | 95.07 | |  |
| **14** | JFD00243 | D831 | 3.05 | 30.27 | | L694, F669, A719, T766, Q767, M769, P770, G772, L820 |
| **15** | BTB11140 | - | | - | | F669, V702, A719, K721, T766, Q767, M769, R817, L820, T830, D831 |
| **16** | NPA016333 | T830 | 2.91 | 99.16 | | L694, V702, A719, K721, E738, M742, L764, T766, P770, F771, G772, D776, G780, L820 |
|  |  | D831 | 3.06 | 35.12 | |  |
| **17** | NPA030739 | C773 | 3.27 | 170.20 | | L694, G695, F669, V702, A719, K721 M769, F771, G772, D776, T777, G780, H781, R817, L820, D831 |
