# Supplementary material for: Unraveling potential EGFR kinase inhibitors: Computational screening, molecular dynamics insights, and MMPBSA analysis for targeted cancer therapy development
Source: PLoS One. 2025 May 9;20(5):e0321500. doi: 10.1371/journal.pone.0321500 (PMC12064201; doi:10.1371/journal.pone.0321500)
Supplement: S4 Table — (DOCX) [file pone.0321500.s005.docx]

**S4 Table.** Summary of 1XKK residues interacting with the ligands

| **S. No.** | **Ligand ID** | **H-bond Interaction** | | | | **Hydrophobic Interaction** |
| --- | --- | --- | --- | --- | --- | --- |
|  |  | **Residue** | **Distance (Å)** | | **Angle** |  |
| **1** | BTB13628 | T830 | 3.25 | | 86.17 | L694, V702, A719, I720, K721, M742, L753, L764, T766, F771, G772, C773, D776, T777, G780, L820, D831, F973, T974, L977 |
| **2** | BTB13627 | T830 | 3.29 | | 84.23 | L694, V702, A719, I720, K721, M742, L753, L764, T766, F771, G772, C773, D776, T777, G780, L820, D831, F973, T974, L977 |
| **3** | NPA032595 | L694 | 3.05 | | 69.50 | V702, A719, K721, T766, L768, F771, G772, C773, D776, G780, N818, L820, T830, D831, F973, T974, L977 |
| **4** | BTB11079 | - | | | - | L694, V702, K721, L768, M769, F771, G772, C773, D776, T777, L820, T830, D831, T974, L977 |
| **5** | JFD00243 | - | | | - | V702, K721, T766, L768, M769, G772, C773, D776, R779, R817, L820, T830, D831 |
| **6** | NPA015124 | L694 | 3.03 | | 124.14 | V702, A719, I720, K721, T766, L768, F771, G772, D776, G780, N818, L820, T830, D831, F973, T974, L977 |
| **7** | NPA027669 | A698 | 3.19 | | 25.27 | L694, G697, F669, V702, A719, K721, L768, M769, G772, C773, D813, R817, N818, L820, T830 |
|  |  | D831 | 2.77 | | 172.72 |  |
| **8** | MBX048666 | M769 | 3.10 | | 16.41 | L694, S696, G697, V702, A719, K721, M742, C751, R752, L753, T766, L768, G772, L820, T830, F832, L834 |
|  |  | D831 | 2.87 | | 120.42 |  |
| **9** | NPA007259 | D831 | 3.33 | | 81.55 | L694, V702, A719, K721, T766, L768, G772, R817, N818, L820, T830, F973, D776, G780, T974, L977 |
| **10** | NPA030938 | K721 | 3.30 | | 38.16 | L694, G695, V702, A719, L753, L764, T766, L768, G772, D776, L820, D831, L977 |
|  |  | Q767 | 3.05 | | 148.66 |  |
|  |  | M769 | 3.05 | | 3.46 |  |
| **11** | ZINC000014241511 | T830 | 2.95 | | 43.58 | L694, G695, V702, A719, K721, M742, C751, R752, L753, L764, T766, L820, D831, F832, L834 |
| **12** | ZINC000008299978 | - | | | - | L694, V702, A719, K721, M742, L753, L764, T766, G772, C773, L820, T830, D831, L977 |
| **13** | ZINC000257243713 | M769 | | 3.00 | 19.03 | G697, V702, A719, I720, K721, C751, R752, L753, L764, T766, G772, R817, L820, D831, F832 |
|  |  | T830 | | 2.67 | 49.38 |  |
| **14** | ZINC000035482583 | D831 | | 3.01 | 129.41 | L694, V702, A719, K721, M742, C751, R752, L753, L764, T766, L768, M769, G772, L820, T830, L834 |
| **15** | ZINC000033088664 | - | | | - | L694, V702, A719, K721, M742, C751, R752, L753, T766, L768, M769, R817, L820, T830, D831, F832, L834 |
