# Supplementary material for: Unraveling potential EGFR kinase inhibitors: Computational screening, molecular dynamics insights, and MMPBSA analysis for targeted cancer therapy development
Source: PLoS One. 2025 May 9;20(5):e0321500. doi: 10.1371/journal.pone.0321500 (PMC12064201; doi:10.1371/journal.pone.0321500)
Supplement: S3 Fig — (DOCX) [file pone.0321500.s007.docx]

**S3 Fig.** **Two-dimensional interactions of top 15 compounds with the active site of inactive EGFR protein (PDB ID: 1XKK).** Residues involved in the formation of hydrogen bonds are depicted as lines, and the hydrogen bonds are illustrated as dotted green lines, with their distances represented in Angstrom units. Hydrophobic interactions between ligands and EGFR amino acid residues are symbolized by red, spoked arcs. Oxygen is denoted by red, nitrogen by blue, and carbon by black circles.

| 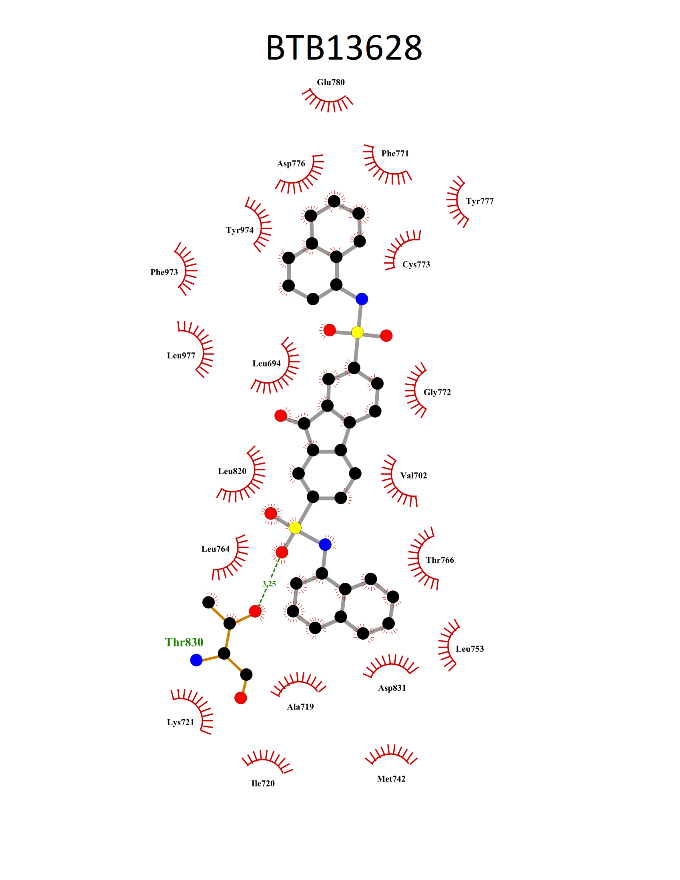 | 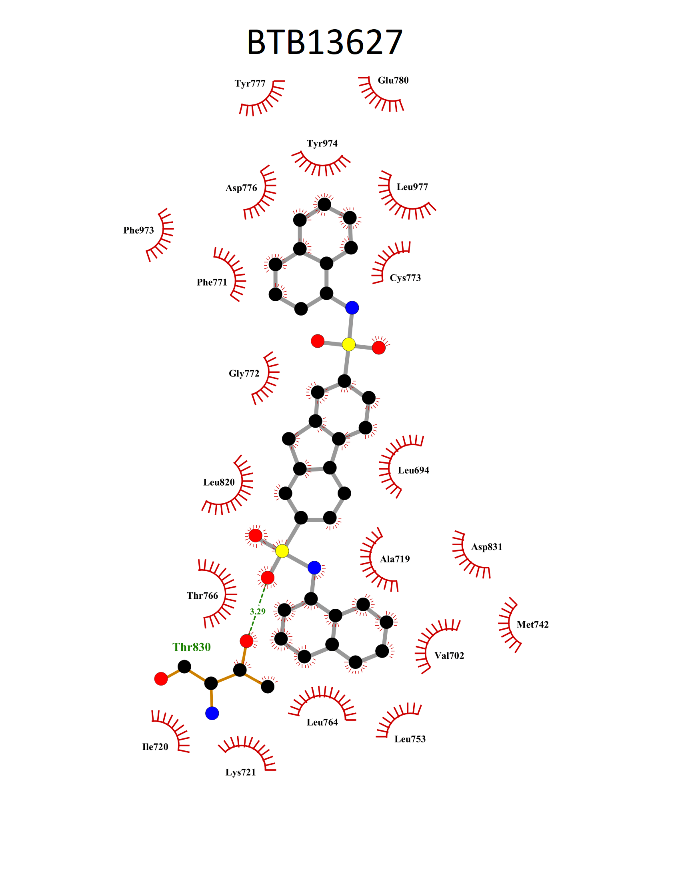 |
| --- | --- |
| 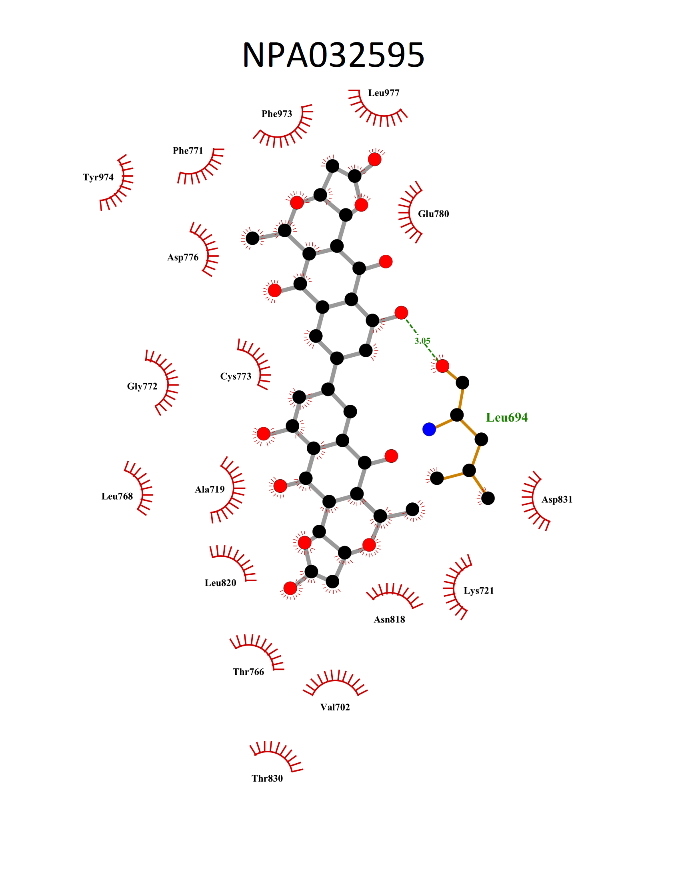 | 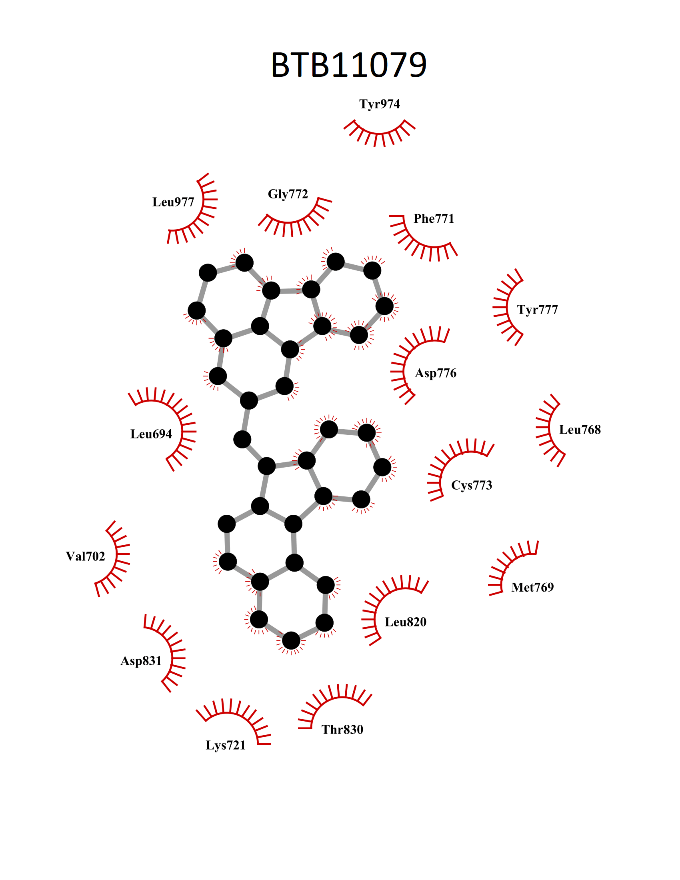 |
| 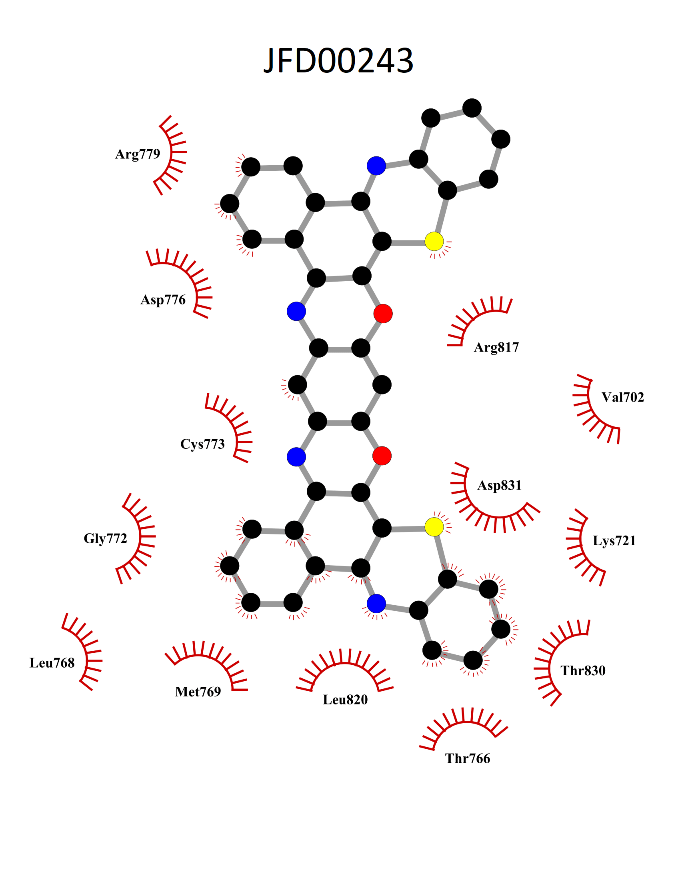 | 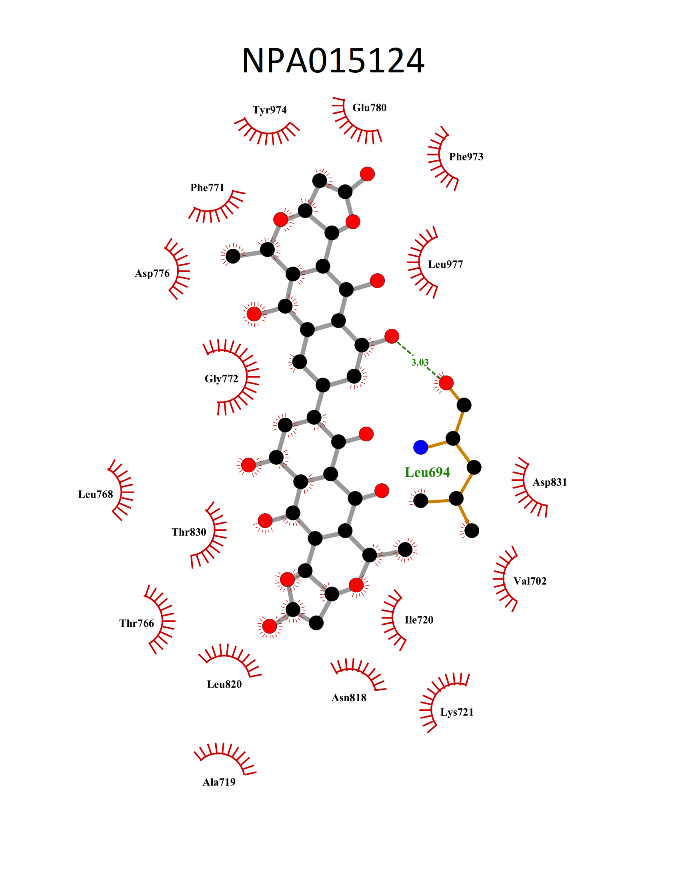 |
| 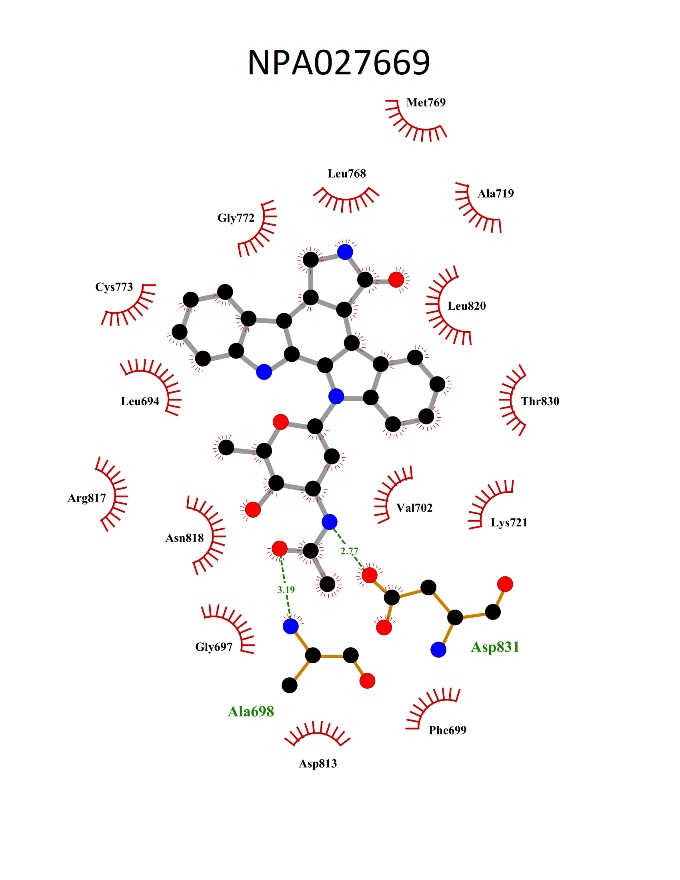 | 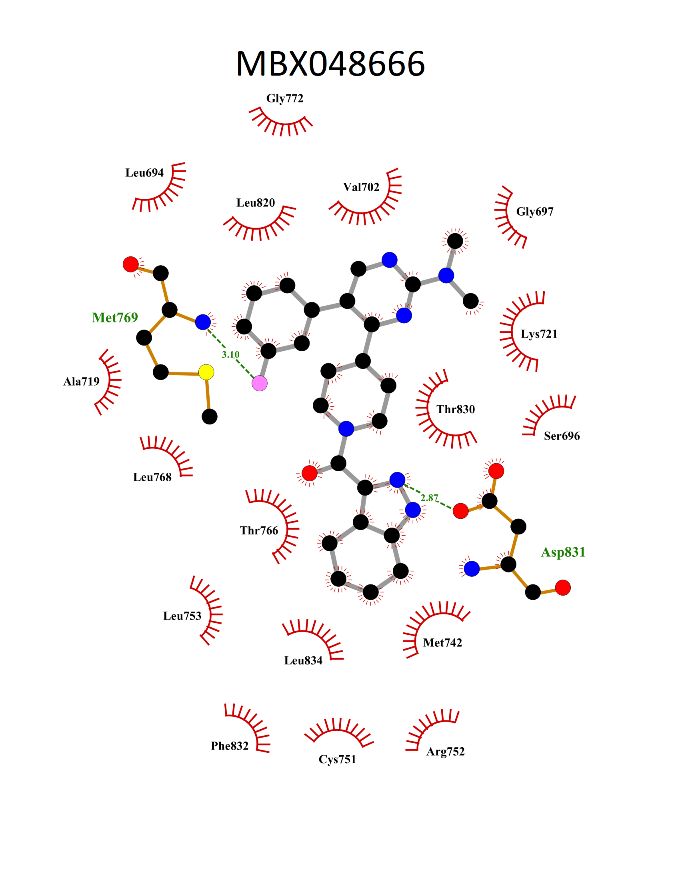 |
| 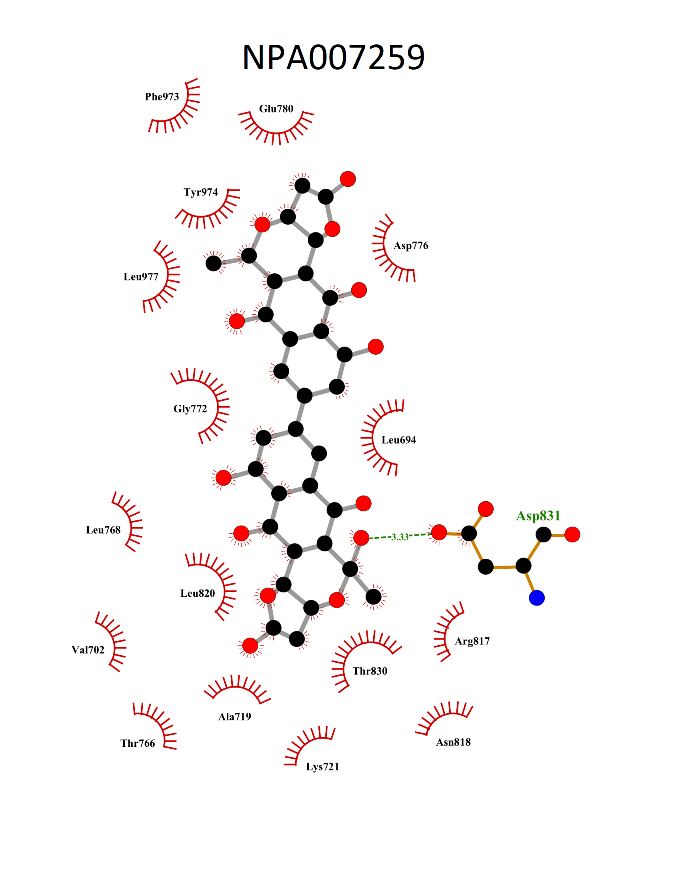 | 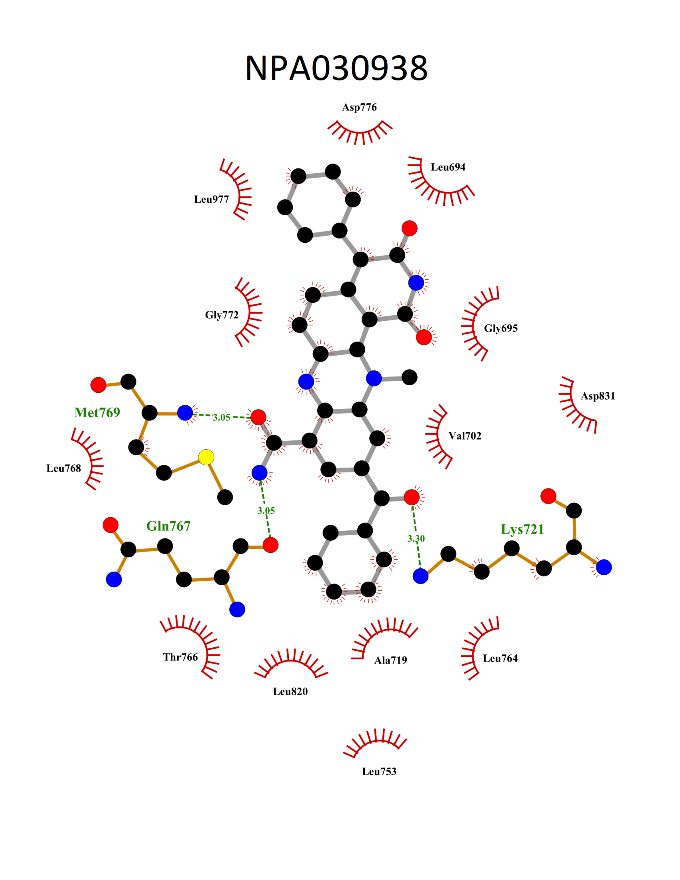 |
| 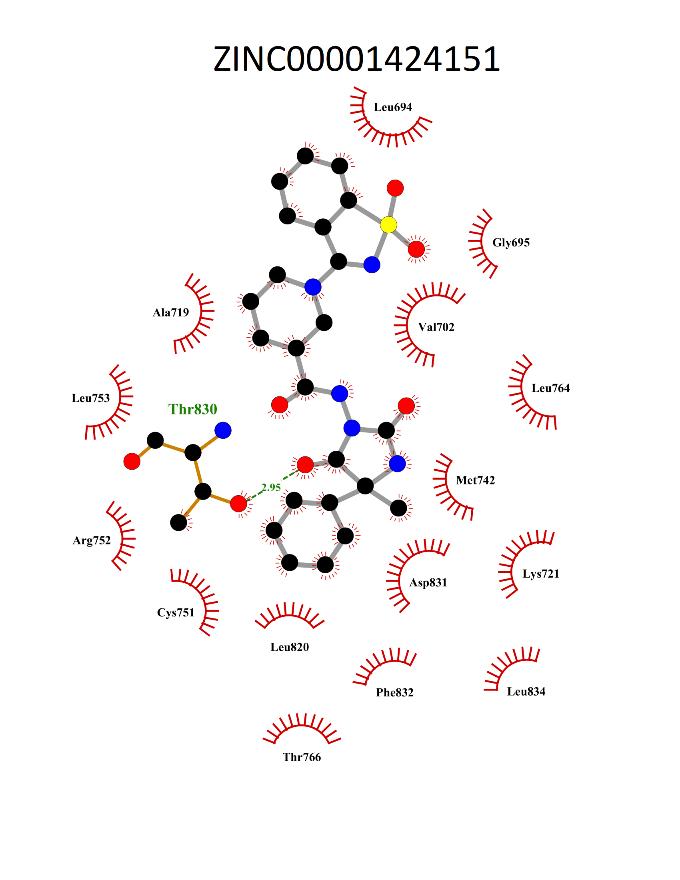 | 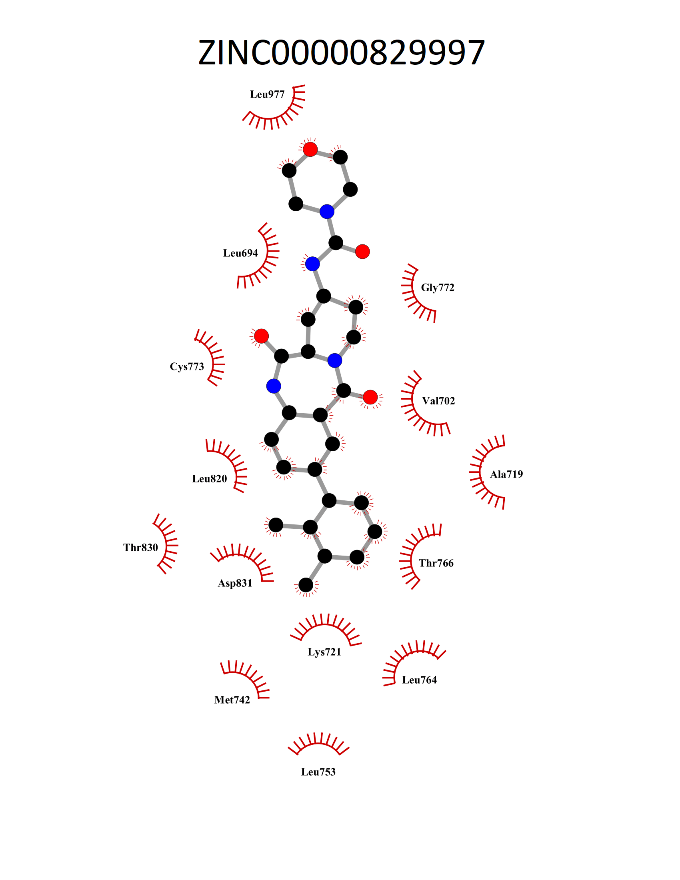 |
| 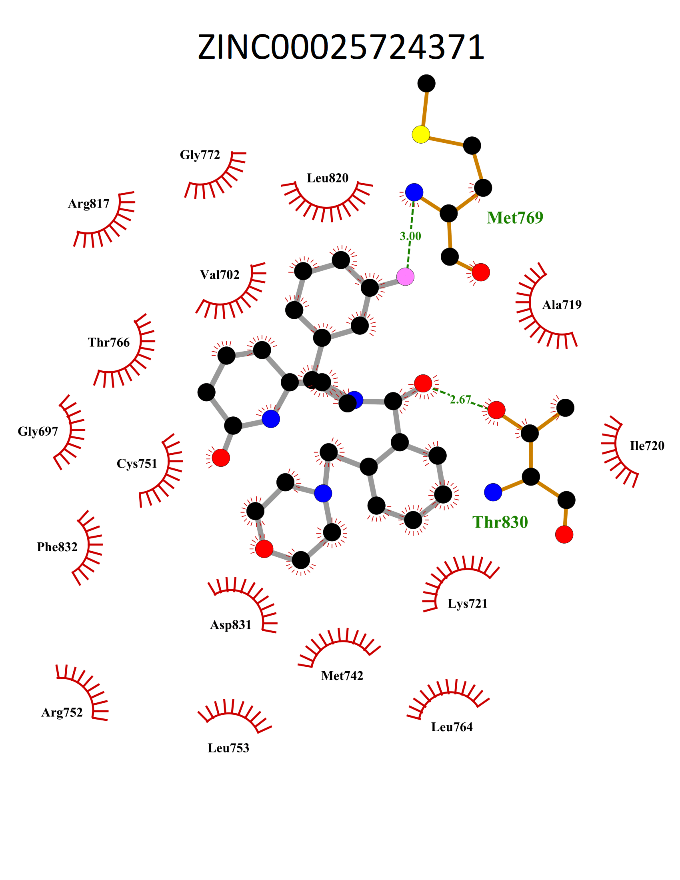 | 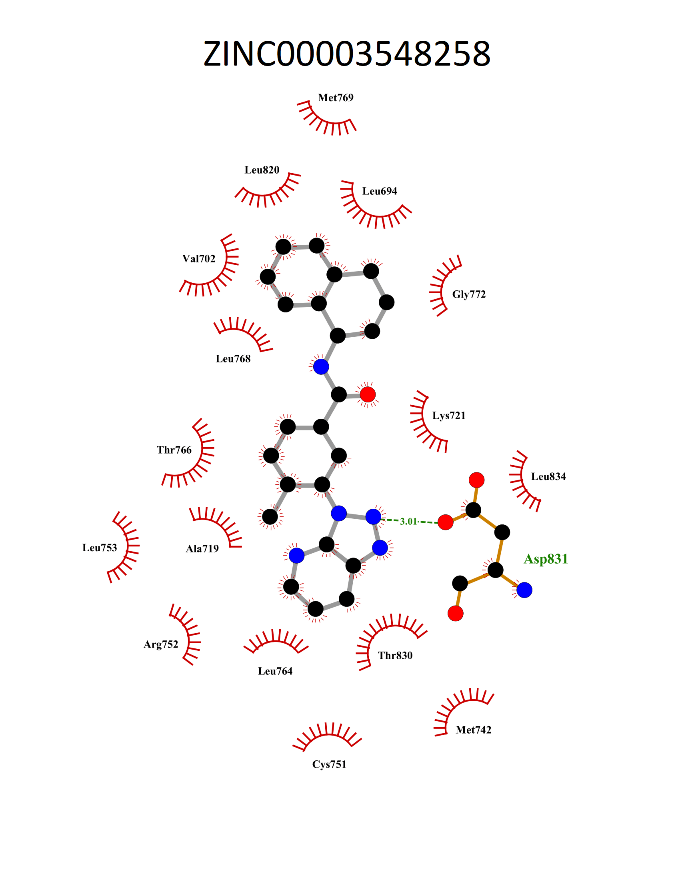 |
| 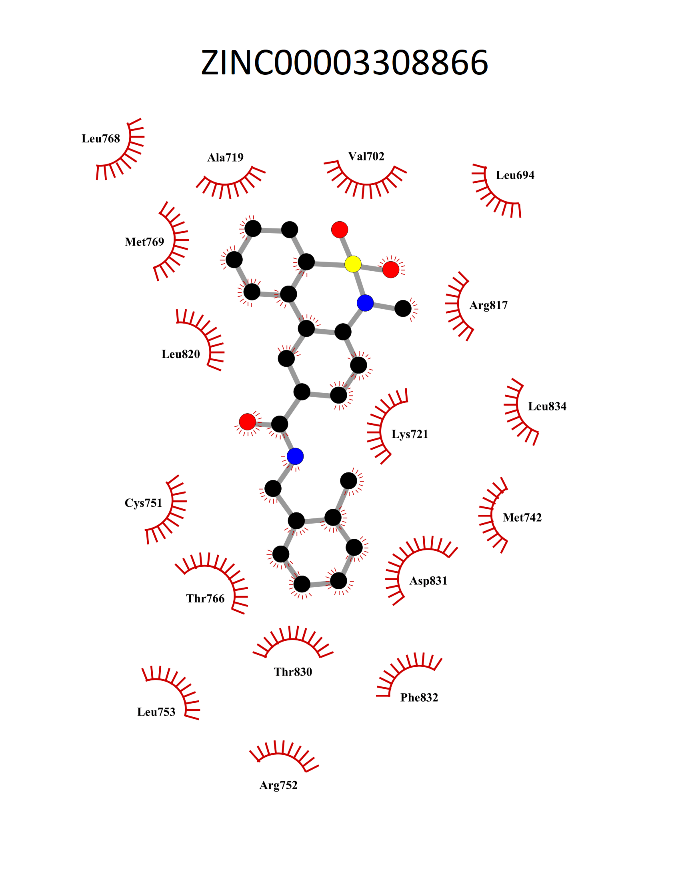 |  |
