# Supplementary figures and images for: Unraveling potential EGFR kinase inhibitors: Computational screening, molecular dynamics insights, and MMPBSA analysis for targeted cancer therapy development
Source: PLoS One. 2025 May 9;20(5):e0321500. doi: 10.1371/journal.pone.0321500 (PMC12064201; doi:10.1371/journal.pone.0321500)

**S4 Fig.** The RMSD graphs for the ligands fit on 1M17 protein


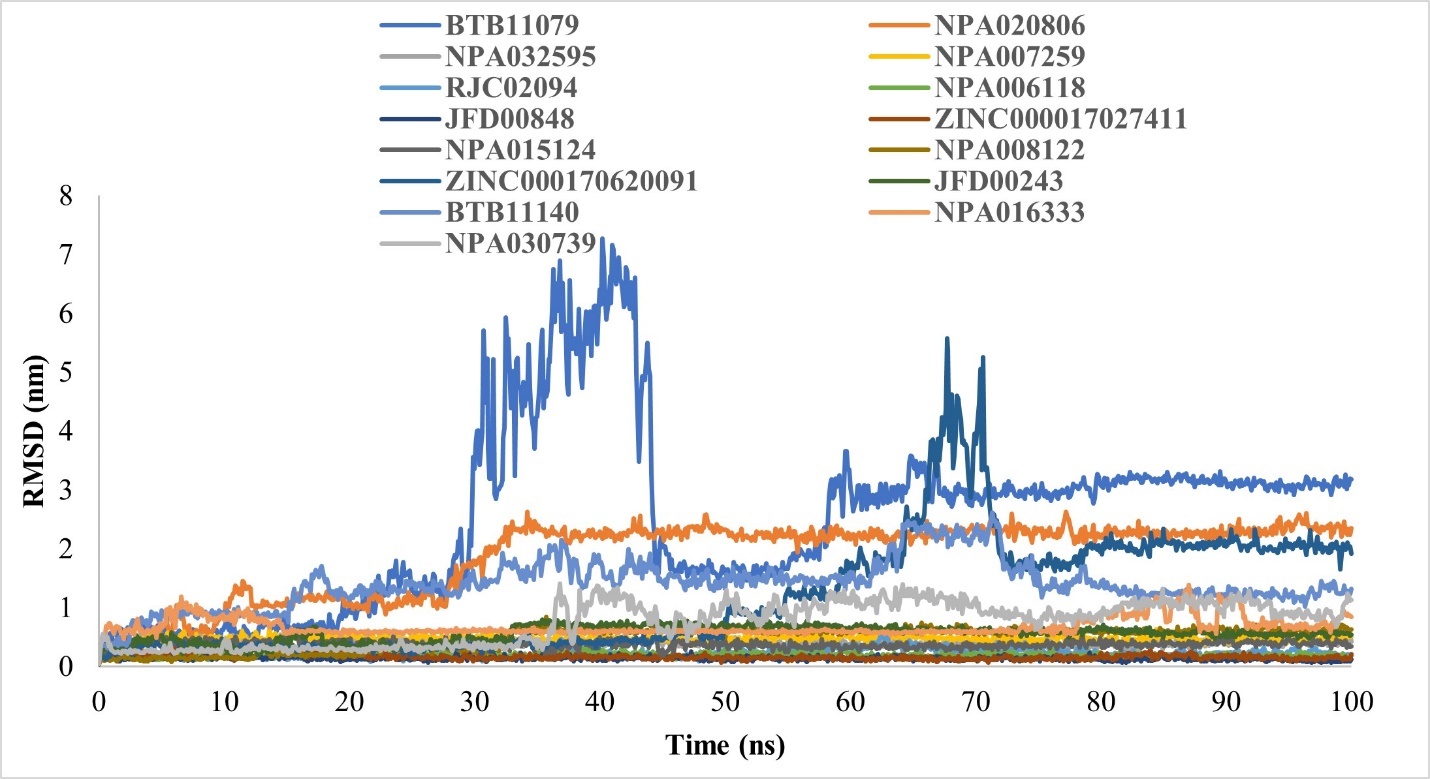

Supplement: S4 Fig — (DOCX) [file pone.0321500.s008.docx]

**S5 Fig.** The RMSD graphs for the ligands fit on 1XKK protein


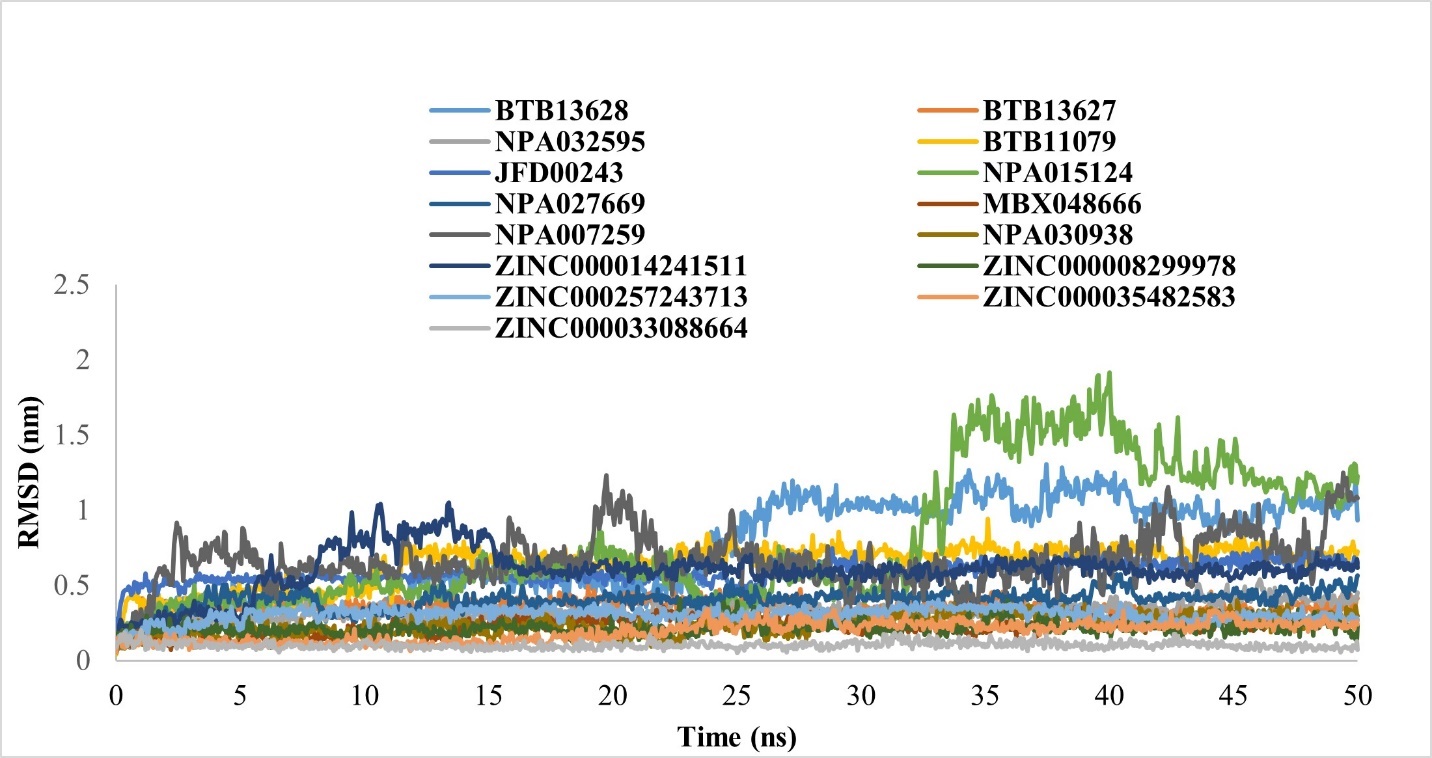

Supplement: S5 Fig — (DOCX) [file pone.0321500.s009.docx]
