# Supplementary material for: Unraveling potential EGFR kinase inhibitors: Computational screening, molecular dynamics insights, and MMPBSA analysis for targeted cancer therapy development
Source: PLoS One. 2025 May 9;20(5):e0321500. doi: 10.1371/journal.pone.0321500 (PMC12064201; doi:10.1371/journal.pone.0321500)
Supplement: S5 Table — (DOCX) [file pone.0321500.s010.docx]

**S5 Table.** Binding free energies (in kcal/mol) for the 1M17-Ligand and 1XKK-Ligand complexes calculated using MM/PBSA

| **Ligand-1M17 Complex** | **Binding Energy kcal/mol** |  | **Ligand-1XKK Complex** | **Binding Energy kcal/mol** |
| --- | --- | --- | --- | --- |
| BTB11079 | -9.708135 |  | BTB13628 | -17.301527 |
| NPA020806 | -9.277495 |  | BTB13627 | -32.50503 |
| NPA032595 | -17.5719 |  | NPA032595 | -20.744253 |
| NPA007259 | -15.989307 |  | BTB11079 | -21.117091 |
| RJC02094 | -22.213872 |  | JFD00243 | -25.914074 |
| NPA006118 | -10.110112 |  | NPA015124 | -11.379101 |
| JFD00848 | -19.431316 |  | NPA027669 | -9.215634 |
| ZINC000017027411 | -16.095115 |  | MBX048666 | -9.017392 |
| NPA015124 | -22.669113 |  | NPA007259 | -10.06927 |
| NPA008122 | -20.848867 |  | NPA030938 | -24.407434 |
| ZINC000170620091 | -11.763882 |  | ZINC000014241511 | 22.226531 |
| JFD00243 | -32.161569 |  | ZINC000008299978 | -21.651151 |
| BTB11140 | -12.26928 |  | ZINC000257243713 | -28.23112 |
| NPA016333 | -18.063206 |  | ZINC000035482583 | -18.846621 |
| NPA030739 | -18.281034 |  | ZINC000033088664 | -27.591012 |
