# Supplementary material for: Unraveling potential EGFR kinase inhibitors: Computational screening, molecular dynamics insights, and MMPBSA analysis for targeted cancer therapy development
Source: PLoS One. 2025 May 9;20(5):e0321500. doi: 10.1371/journal.pone.0321500 (PMC12064201; doi:10.1371/journal.pone.0321500)

**S6 Fig.** Per-residue energy decomposition analysis from MMPBSA showing the contribution of individual residues to the total binding energy of **A)** 1M17–ATP, **B)** 1M17–JFD00243, **C)** 1M17–NPA015124, **D)** 1M17–RJC02094, **E)** 1M17–NPA008122, **F)** 1M17–JFD00848, **G)** 1M17–Erlotinib, **H)** 1XKK–BTB13627, **I)** 1XKK–ZINC000257243713, **J)** 1XKK–ZINC000033088664, **K)** 1XKK–JFD00243, and **L)** 1XKK–NPA030938 complexes, respectively.


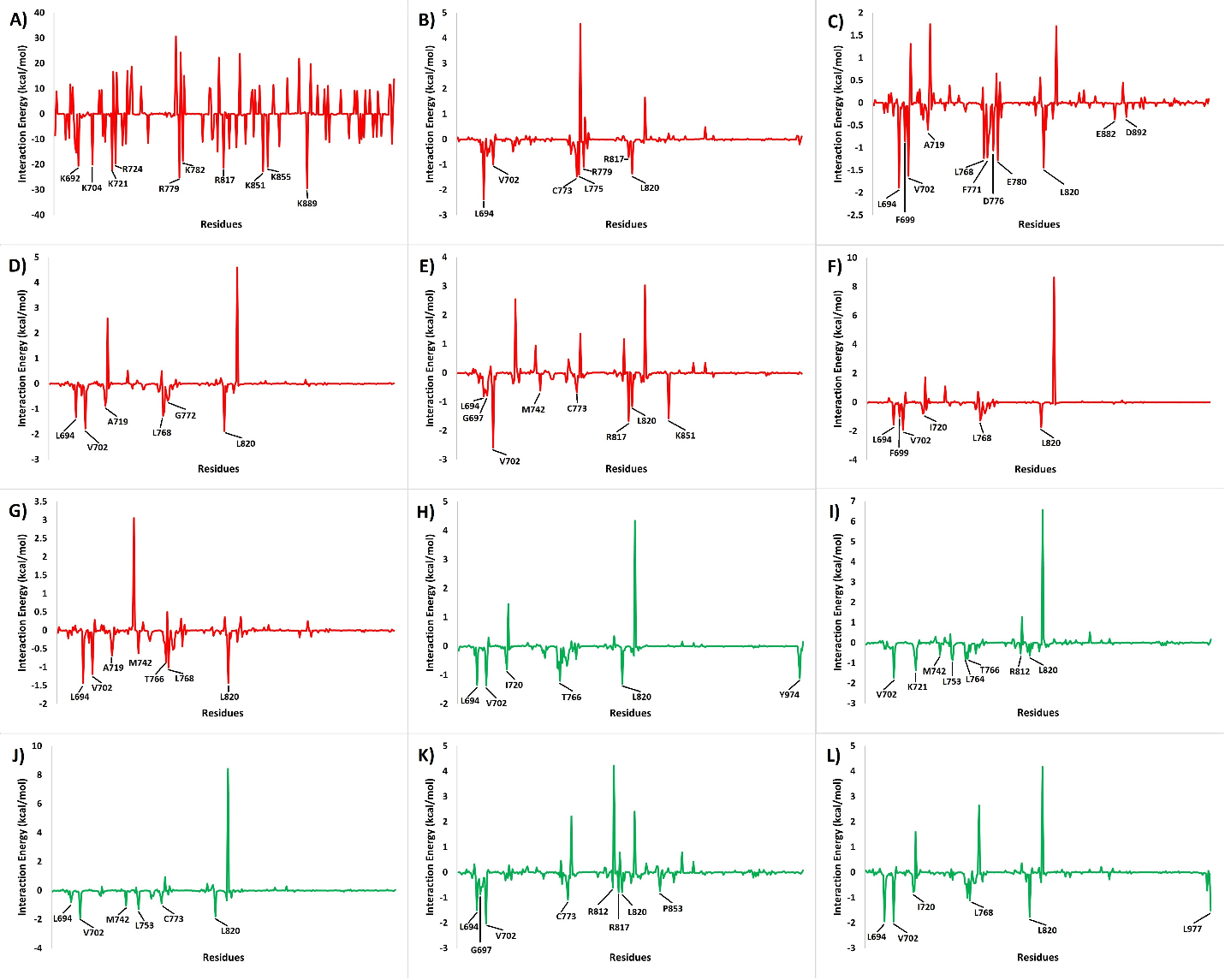

Supplement: S6 Fig — (DOCX) [file pone.0321500.s011.docx]
