# Supplementary material for: Unraveling potential EGFR kinase inhibitors: Computational screening, molecular dynamics insights, and MMPBSA analysis for targeted cancer therapy development
Source: PLoS One. 2025 May 9;20(5):e0321500. doi: 10.1371/journal.pone.0321500 (PMC12064201; doi:10.1371/journal.pone.0321500)
Supplement: S6 Table — (DOCX) [file pone.0321500.s012.docx]

**S6 Table.** Pharmacokinetic profile of screened compounds

| **RJC02094** | **JFD00848** | **NPA015124** | **NPA008122** | **JFD00243** | **BTB13627** | **NPA030938** | **ZINC000257243713** | **ZINC000033088664** |  |
| --- | --- | --- | --- | --- | --- | --- | --- | --- | --- |
| 76.34 | 76.34 | 200.03 | 117.85 | 118.50 | 109.10 | 127.91 | 61.88 | 74.86 | **TPSA**  **(Å)** |
| 3.28 | 3.38 | 1.03 | 1.82 | 7.50 | 5.76 | 3.38 | 2.83 | 3.43 | **Consensus Log *P*_o/w_** |
| Moderate | Moderate | Moderate | Moderate | Poor | Poor | Moderate | Soluble | Moderate | **LogS**  **ESOL** |
| Yes | Yes | No | No | No | No | No | Yes | Yes | **BBB** |
| High | High | Low | High | Low | Low | Low | High | High | **GI absorption** |
| No | No | Yes | Yes | Yes | No | No | Yes | No | **P-gp substrate** |
| No | No | No | No | No | Yes | No | No | Yes | **CYP1A2 inhibitor** |
| Yes | Yes | No | No | No | No | Yes | No | Yes | **CYP2C19 inhibitor** |
| Yes | Yes | No | Yes | No | No | Yes | Yes | Yes | **CYP2C9 inhibitor** |
| Yes | Yes | No | Yes | No | No | Yes | Yes | Yes | **Lipinski** |
| 0.55 | 0.55 | 0.17 | 0.55 | 0.17 | 0.17 | 0.55 | 0.55 | 0.55 | **Bioavailability Score** |
| 0 | 0 | 0 | 0 | 0 | 0 | 0 | 0 | 0 | **PAINS** |
| 4.46 | 4.58 | 5.79 | 5.23 | 5.08 | 3.89 | 3.48 | 4.33 | 3.43 | **Synthetic accessibility** |

**TPSA**: Topological Polar Surface Area, **ESOL**: Estimated solubility, **BBB**: Blood-Brain Barrier, **PAINS**: Pan Assay Interference Structures
